# CNS axonal degeneration and transport deficits at the optic nerve head precede structural and functional loss of retinal ganglion cells in a mouse model of glaucoma

**DOI:** 10.1186/s13024-020-00400-9

**Published:** 2020-08-27

**Authors:** Prabhavathi Maddineni, Ramesh B. Kasetti, Pinkal D. Patel, J. Cameron Millar, Charles Kiehlbauch, Abbot F. Clark, Gulab S. Zode

**Affiliations:** Department of Pharmacology and Neuroscience and the North Texas Eye Research Institute, IREB-535, University of North Texas Health Science Center, 3500 Camp Bowie Blvd, Fort Worth, TX 76107 USA

**Keywords:** Neurodegeneration, Ocular hypertension, Mouse model of glaucoma, Glucocorticoid-induced glaucoma, POAG, Retinal ganglion cell loss, Optic nerve degeneration, Trabecular meshwork, Intraocular pressure, Anterograde transport deficits, And optic nerve head axonal degeneration

## Abstract

**Background:**

Glaucoma is a leading neurodegenerative disease affecting over 70 million individuals worldwide. Early pathological events of axonal degeneration and retinopathy in response to elevated intraocular pressure (IOP) are limited and not well-defined due to the lack of appropriate animal models that faithfully replicate all the phenotypes of primary open angle glaucoma (POAG), the most common form of glaucoma. Glucocorticoid (GC)-induced ocular hypertension (OHT) and its associated iatrogenic open-angle glaucoma share many features with POAG. Here, we characterized a novel mouse model of GC-induced OHT for glaucomatous neurodegeneration and further explored early pathological events of axonal degeneration in response to elevated IOP.

**Methods:**

C57BL/6 J mice were periocularly injected with either vehicle or the potent GC, dexamethasone 21-acetate (Dex) once a week for 10 weeks. Glaucoma phenotypes including IOP, outflow facility, structural and functional loss of retinal ganglion cells (RGCs), optic nerve (ON) degeneration, gliosis, and anterograde axonal transport deficits were examined at various stages of OHT.

**Results:**

Prolonged treatment with Dex leads to glaucoma in mice similar to POAG patients including IOP elevation due to reduced outflow facility and dysfunction of trabecular meshwork, progressive ON degeneration and structural and functional loss of RGCs. Lowering of IOP rescued Dex-induced ON degeneration and RGC loss, suggesting that glaucomatous neurodegeneration is IOP dependent. Also, Dex-induced neurodegeneration was associated with activation of astrocytes, axonal transport deficits, ON demyelination, mitochondrial accumulation and immune cell infiltration in the optic nerve head (ONH) region. Our studies further show that ON degeneration precedes structural and functional loss of RGCs in Dex-treated mice. Axonal damage and transport deficits initiate at the ONH and progress toward the distal end of ON and target regions in the brain (i.e. superior colliculus). Most of anterograde transport was preserved during initial stages of axonal degeneration (30% loss) and complete transport deficits were only observed at the ONH during later stages of severe axonal degeneration (50% loss).

**Conclusions:**

These findings indicate that ON degeneration and transport deficits at the ONH precede RGC structural and functional loss and provide a new potential therapeutic window for rescuing neuronal loss and restoring health of damaged axons in glaucoma.

## Background

Glaucoma is a heterogeneous group of optic neuropathies characterized by progressive loss of retinal ganglion cells (RGCs) and optic nerve degeneration that results in irreversible vision loss [1]. According to 2013 global estimates, 64.3 million people (aged 40 to 80 years) have glaucoma, and this number is estimated to reach 111.8 million by 2040 [2, 3] imposing a major threat to global health [4]. Primary open-angle glaucoma (POAG) is the most common form of glaucoma, accounting for at least 74% of all cases [5]. Elevated intraocular pressure (IOP) is the most important and only known modifiable risk factor associated with POAG [6–8]. Under normal conditions, IOP is tightly regulated via balance of aqueous humor (AH) production by the ciliary body and its outflow through trabecular meshwork (TM) tissue [9, 10]. Increased resistance to AH outflow due to TM dysfunction is responsible for IOP elevation in POAG [11, 12]. The glaucomatous damage is associated with characteristic biochemical and morphological changes in the TM [13, 14]. Chronic IOP elevation leads to glaucomatous neurodegeneration including loss of RGCs and optic nerve degeneration, resulting in irreversible vision loss [12]. Despite therapeutic reduction of IOP, vision loss still continues to progress in most glaucoma patients [15–17]. Therefore, there is a critical need to understand early pathological events of glaucomatous neurodegeneration, which can be effectively targeted along with IOP lowering treatments to prevent vision loss.

In order to understand the pathophysiology of glaucomatous neurodegeneration and to develop targeted neuroprotective agents, it is essential to develop simple and reliable animal models that can faithfully replicate human POAG. Since the structural and functional anatomy of the conventional outflow pathway and AH outflow dynamics are similar between human and mouse eyes [18], several mouse models of ocular hypertension (OHT) have been developed. These mouse models include spontaneous mutant DBA/2 J mice and inducible mouse models that elevate IOP via physical blockage of AH outflow through the TM or block the pupil to prevent inflow of AH into the anterior chamber [19–24]. These models exhibit RGC cell death and optic nerve degeneration and have been instrumental in providing valuable mechanistic insights of glaucomatous neurodegeneration. However, several aspects of glaucoma cannot be studied in these models. Since most of these models elevate IOP by physically blocking outflow pathway [21–23], they do not faithfully reproduce human POAG conditions in which the outflow pathway is open. Silicon oil induces OHT by blocking the pupil which prevents inflow of AH into the anterior chamber without damaging any ocular tissues and leads to secondary open angle glaucoma. It is likely that the pathophysiology of glaucomatous neurodegeneration due to chronic IOP elevation observed in POAG is different than these established models and these human POAG pathways can be explored more effectively utilizing mouse models that mimic POAG. Therefore, it is necessary to develop a novel mouse model of chronic and mild OHT-induced glaucomatous neurodegeneration that mimics human POAG.

OHT is a serious adverse effect of glucocorticoid (GC) therapy [18, 25–27], and GCs are widely used to treat various inflammatory and immune mediated systemic, local, and ocular diseases [18]. Topical or systemic administration of potent GCs (such as dexamethasone or betamethasone) for 4 to 6 weeks cause OHT in approximately 30 to 40% of the general population [28, 29]. If left untreated, prolonged GC-induced OHT can lead to glaucomatous optic neuropathy and vision loss [25]. GC responsiveness is significantly higher in POAG patients. Strikingly, the morphological and clinical features of GC-induced OHT and its associated iatrogenic open-angle glaucoma closely resembles POAG [29–31]. Similar to POAG, GCs also cause morphological and biochemical changes in the TM including extracellular matrix (ECM) remodeling [32–34], altered cytoskeleton changes [35] and reduced phagocytic activity [36].

Since GC-induced OHT and its associated iatrogenic open-angle glaucoma share many features of POAG, several laboratories have attempted to develop mouse models of GC-induced glaucoma [37–39]. Most of these studies were limited to GC-induced OHT and outflow facility obstructions, however IOP elevation induced in these models is mild and may not be sufficient to cause glaucomatous neurodegeneration. We have recently developed a simple and reproducible mouse model of GC-induced OHT using periocular injections of dexamethasone-21-acetate (Dex) [40]. It is important to determine whether Dex-induced OHT is sustained and sufficient to develop glaucomatous neurodegeneration, similar to human POAG. Here, we show that prolonged Dex treatment leads to IOP dependent glaucomatous neurodegeneration including significant structural and functional loss of RGCs as well as optic nerve degeneration. Using this model, we further sought to study early pathological events of optic nerve degeneration in response to elevated IOP. We show that RGC soma and most of the anterograde transport mechanism persist despite initial optic nerve degeneration. RGC soma loss and complete transport deficits were observed only after severe optic nerve degeneration.

## Materials and methods

### Experimental animals

Three-month old male C57BL/6 J mice obtained from the Jackson Laboratory (Bar Harbor, ME) were used in our study. Animals were fed standard chow ad libitum and housed under controlled conditions of temperature (21 °C to 26 °C), humidity (40 to 70%) and maintained on a 12 h light/12 h dark cycle. The number of mice utilized in each experiment is denoted in the representative figures or figure legends. The experimental protocol was approved by the Institutional Animal Care and Use Committee (IACUC) of the University of North Texas Health Science Center (UNTHSC) (Protocol #: IACUC-2018-0032). Mice were euthanized by inhalation of carbon dioxide followed by cervical dislocation as approved by institutional IACUC protocol.

### Periocular administration of Dex

Dex suspension was prepared by mixing 10 mg of micronized Dex-Acetate (Spectrum Chemicals, New Brunswick, NJ) in 1 mL of vehicle (Veh) suspension. Ingredients and preparation of vehicle suspension was described previously [40]. A uniform suspension with desired Dex particle size was achieved by mixing the suspension along with two stainless steel 5-mm beads (Qiagen, Valencia, CA) in a TissueLyser LT (Qiagen) for 10 min at 50 oscillations/sec and further rotated overnight at 4 °C until use. Mice under anesthetic conditions (isoflurane (2.5%); oxygen (0.8 L/min)) were injected weekly with 20 μL/eye of either Veh or freshly made Dex (i.e. 200 μg) suspension via the periocular route using a 32-gauge needle attached to a Hamilton glass micro syringe. For most experiments both eyes were injected with either Veh or Dex (bilateral). To study axonal transport, one eye was injected with Veh while the contralateral eye was injected with Dex.

### Intraocular pressure measurements

IOPs were measured under anesthetic conditions (isoflurane (2.5%); oxygen (0.8 L/min)) using a TonoLab rebound tonometer (Colonial Medical Supply, Franconia, NH). Both day and night-time IOPs were monitored once a week throughout the treatment periods. IOP measurements were recorded in a masked manner and an average of six IOP readings were taken at each time period. In addition, dark-adopted night-time IOPs were performed as described previously [41]. IOP measurements were completed within 4 min in order to avoid the effect of isoflurane on IOP.

### Aqueous humor outflow facility

Outflow facilities in Veh and Dex-injected mice were measured using the constant-flow infusion technique as described previously [42]. Briefly, mice were anesthetized with ketamine/xylazine solution (100/10 mg/kg) and body temperature was maintained at physiological levels by placing mice on a 37 °C heating pad during the entire procedure. Topical ocular proparacaine HCl (0.5%) eye drops were applied to both eyes for corneal anesthesia. Anterior chambers were cannulated with a 30-gauge needles through cornea without touching the iris, anterior lens capsule or corneal endothelium. The cannulated needles were connected to a pre-calibrated flow-through BLPR-2 pressure transducer and maintained continuous pressure within the perfusion system. The opposing end of the transducer was connected to a 1 mL syringe loaded into a microdialysis infusion pump. The entire perfusion system was filled with sterile phosphate buffer solution (PBS). Eyes were infused at a flow rate of 0.1 μL/min to 0.5 μL/min (in 0.1 μL/min increments), and three stabilized pressures at 5 min intervals were recorded for each flow rate. An average of these three stabilized pressures was considered as a mean stabilized pressure. Aqueous humor outflow facility was calculated as the reciprocal of the slope of a plot of mean stabilized pressure as the ordinate and flow rate as the abscissa.

### Mouse slit-lamp examination

Ocular abnormalities and inflammation in Veh and Dex-injected eyes were examined using slit-lamp microscopy (SL-D7; Topcon) and photo-documented with a digital camera (D100; Nikon).

### Fixing of ocular and extra-ocular tissues via intracardiac perfusion

Mice were subjected to intracardiac perfusion of PBS followed by 4% paraformaldehyde (PFA) in order to fix ocular and/or extra-ocular tissues. Briefly, mice were weighed and then anesthetized with an injectable anesthetic cocktail (ketamine 100 mg/kg; xylazine 10 mg/kg; acepromazine 3 mg/kg (administered I.P., 1 mL/100 g)). Absence of flexor withdrawal response, a tail pinch, and the palbebral (blink) reflex was assessed and then intracardiac perfusion was performed. After intracardiac perfusion, absence of respiration and heartbeat was confirmed, and a unilateral thoracotomy was performed. Following the thoracotomy, ocular and/or extra-ocular tissues were dissected carefully and fixed in 4% PFA for 12 h at 4 °C.

### Immunostaining

As described previously [43], paraffin embedded mouse or human tissue sections were deparaffinized in xylene and rehydrated gradually by washing with 100, 95, 70, 50% ethanol for 5 min in each. Antigen retrieval was performed by incubating the sections in citrate buffer (pH 6.0) at 100 °C for 13 min, followed by an incubation with citrate buffer (pH 6.0) for 30 min at room temperature. For OCT embedded cryosections, sections without antigen retrieval were incubated directly in blocking buffer (PBS containing 10% goat serum and 0.2% Triton X-100) for 2 h in a dark and humidified chamber. Note that depending on the tissue type, the concentration of Triton X-100 varied from 0.2 to 2%. Tissue sections were washed briefly and incubated overnight with specific primary antibodies at 4 °C. Following 3 washes in PBS, sections were incubated with appropriate secondary antibodies for 2 h at room temperature. Tissue sections were washed with PBS and mounted with mounting medium containing DAPI nuclear stain (Vector Labs, Inc., Burlingame, CA, USA). Images were captured using a Keyence fluorescence microscope (Itasca, IL, USA) and processed in ImageJ [44]. Tissue sections incubated without primary antibody served as a negative control and the relative signal intensities were subtracted with averaged background intensity. All the antibodies used in this study are listed in Table 1.

**Table 1:** List of antibodies used in the study

Primary Antibody	Source	Catalog number	Dilutions factor
Fibronectin (FN)	Santa Cruz Biotechnology	sc-18,825	1 in 200
Collagen1 (ColI)	Novus Biology	NB600–408	1 in 200
Phalloidin 650	Cell Signaling	12956S	1 in 50
Laminin	Sigma	L9393	1 in 200
αSMA	Abcam	ab7817	1 in 200
GFAP	Abcam	ab10062	1 in 100
NF-H	Abcam	ab8135	1 in 100
F4/80	Abcam	ab6640	1 in 200
TNF-alpha	Abcam	ab1793	1 in 100
CoxIV	Abcam	ab16056	1 in 100
RBPMS	Gentex	GTX118619	1 in 200
Cholera Toxin Subunit B (Recombinant), Alexa Fluor™ 555 Conjugate	Thermo Fisher Scientific	C34776	3 μl of 0.1% CTB/eye
Cholera Toxin Subunit B (Recombinant), Alexa Fluor™ 488 Conjugate	Thermo Fisher Scientific	C34775	3 μl of 0.1% CTB/eye
**Secondary Antibody**	**Source**	**Catalog number**	**Dilutions factor**
Goat anti rabbit 568	Invitrogen	A11011	1 in 500
Goat anti rabbit 488	Invitrogen	A11034	1 in 500
Goat anti rabbit 680	Life technologies	A21076	1 in 500
Goat anti mouse 568	Life technologies	A11031	1 in 500
Goat anti mouse 488	Invitrogen	A11001	1 in 500

### Pattern electroretinography

Pattern electroretinography (PERG) was performed to analyze the function of RGCs by measuring amplitudes and latency as described previously [45]. Briefly, mice were anesthetized with ketamine/xylazine solution (100/10 mg/kg) and placed on a heated stage during the entire procedure. PERG measurements were conducted using the Miami PERG system (Jorvec, Miami, Fl) as per manufacturer’s instructions.

### Whole-mount retina staining

Whole-mount retina staining with RBPMS antibody was performed to examine the total number of surviving RGCs in Veh and Dex-injected mice. Briefly, eyes were enucleated from euthanized mice and fixed in 4% PFA for 12 h at 4 °C. After rinsing the eyeball in PBS, the anterior chamber was removed, and retina was carefully separated from the posterior cup. Isolated retinas were incubated in blocking buffer (PBS containing 10% goat serum and 0.2% Triton X-100) for 12 h at 4 °C. Retinas were incubated with RBPMS antibody for 3 days at 4 °C and washed for 2 h in PBS. After washing, retinas were incubated with a corresponding secondary antibody (goat anti-rabbit 568, 1:500; Invitrogen) for 2 h at room temperature, washed for 1 h in PBS and then mounted with mounting medium containing DAPI nuclear stain. For counting RGCs, at least 16 non-overlapping images from the entire retina were captured at 200x magnification using a Keyence fluorescence microscope (Itasca, IL, USA) and RBPMS-positive cells were counted using ImageJ software [44]. To examine reactive astrocytes in the retina, we incubated whole-mount retina with GFAP antibody along with RBPMS.

### Axonal anterograde transport of cholera toxin B

Axonal anterograde transport deficits was analyzed by cholera toxin B (CTB) labelling. Following 5, 8- or 10-weeks post injections, mice were anesthetized and injected intravitreally with 3 μL of 0.1% CTB (reconstituted in PBS) conjugated with either Alexa fluor 555 or Alexa fluor 480 (Invitrogen, Life Technologies, Grand Island, NY, USA) using a Hamilton syringe with a 33-gauge needle. After 48 h, mice were euthanized, and eyes and brains were enucleated and fixed in 4% PFA for 12 h at 4 °C. Fixed eyes and brains were washed in PBS and incubated in gradient concentrations of sucrose solution (10 to 30%) for an additional 3 days, and then embedded in OCT compound and cryo-sectioned at 10 μm thickness. For visualizing the axonal anterograde transport of CTB in the SC, serial frontal sections of brain were taken at 30 μm thickness. All the sections were washed with PBS, mounted with mounting medium containing DAPI nuclear stain and images were captured at the same laser intensities using a Keyence fluorescence microscope.

### Assessment of optic nerve damage by PPD staining and TEM

PPD staining was used to examine the optic nerve degeneration in Veh and Dex-injected mice as described previously [41]. PPD lightly stains the myelin sheath of all healthy axons while the axoplasm of damaged or dying axons is darkly stained. In brief, the optic nerve was separated from the eyeball and fixed overnight in a phosphate-buffered 3% glutaraldehyde/paraformaldehyde mixture at 4 °C. Following overnight treatment in 1% osmium tetroxide at 4 °C, optic nerves were rinsed in 0.1 M phosphate buffer and 0.1 M sodium-acetate buffer, then dehydrated in graded ethanol concentrations. After embedding optic nerves in resin (Eponate-12; Ted Pella), 1 μm sections were cut and stained in 1% PPD for 10 min. Optic nerve axons were counted manually using a previously described method [41]. In short, 10 non-overlapping images covering the entire optic nerve were taken at 1630x magnification, and axons were counted in the area equal to 10% of the total nerve cross-sectional area. To perform TEM, 1 μm sections were processed for TEM analysis as described previously [39].

### Topical ocular eye drops of IOP lowering agents

To reduce Dex-induced OHT, we utilized commercial IOP lowering eye drops, Cosopt (dorzolamide/timolol 22.3/6.8 mg; Valeant Pharmaceuticals North America LLC, Bridgewater, NJ, USA) and 0.005% Latanoprost (Alcon Laboratories, Inc. Fort Worth, TX, USA). Mice were injected bilaterally with periocular injections of Veh and Dex and one group of each Veh or Dex mice received either water as a control eye drops (6 μL/eye) and other group received Cosopt (b.i.d) (6 μL/eye) & Latanoprost (once a day at 5 PM) (6 μL/eye) eye drops throughout the study period starting from the day of injections. Mice were restrained while dosing. IOPs were monitored weekly, and PERG, RGCs labeling and PPD staining of ON was performed after 8 weeks of injections.

### Withdrawal of Dex treatment

To examine the effect of Dex withdrawal on IOP and RGC loss, we discontinued Dex injections in a group of mice as described previously [40]. Briefly, three-month old male C57BL/6 J mice were injected bilaterally once a week for 5 weeks with either Veh or Dex. IOPs were monitored weekly to ensure IOP elevation in Dex-injected mice. After 5 weeks of injections, Dex-injected mice were randomly divided into two groups. Dex treatment was stopped in one group of mice while another group continued to receive Dex for additional 5 weeks. IOPs were monitored weekly and whole mount retinal staining with RBPMS was performed at the end of 10 weeks.

## Results

### Periocular injection of Dex leads to sustained IOP elevation, reduced outflow facility and increased ECM deposition in the mouse TM

**Fig. 1:**
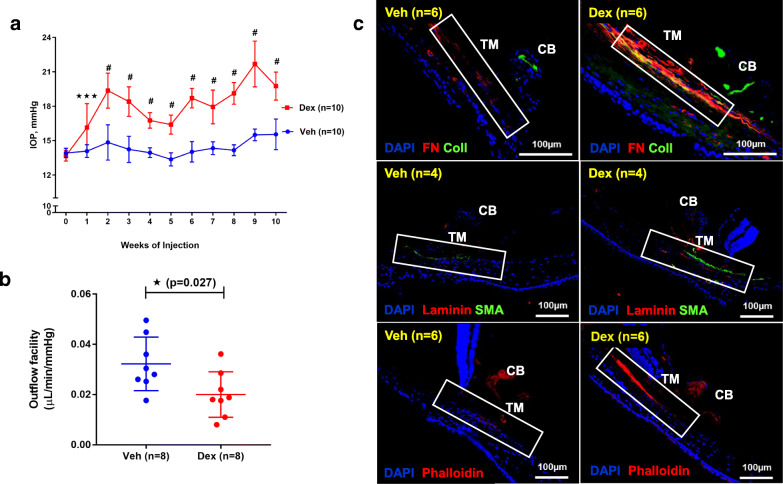
Periocular administration of Dex leads to elevated IOP, reduced aqueous humor outflow facility and increased ECM deposition in mouse TM tissue. **a** C57BL/6 J mice were periocularly injected with either Veh or Dex in both eyes once a week for 10 weeks, and IOPs were monitored weekly. Dex injections lead to sustained and significant IOP elevation. Data are shown as mean ± SD (n = 10 in each group, 2-WAY ANOVA with multiple comparison, ***p = 0.0005, #*p* < 0.0001). **b** Age matched C57BL/6 J mice were injected bilaterally once a week for 4 weeks with either Veh or Dex, and outflow facility was measured using constant flow infusion method. A significant reduction (~ 37.8%) in outflow facility was observed in 4 weeks post Dex-injected mice compared to Veh-injected mice (*n* = 8 in each group, unpaired t-test, two tailed, mean ± SD, *p = 0.027). **c** Anterior segment tissues after 10 weeks of Veh (left panel) and Dex (right panel) injected mice were immunostained with ECM markers including fibronectin (FN), collagen I (ColI) and laminin along with α-smooth muscle actin (SMA) or phalloidin. Iridocorneal angles are represented by a rectangle white box in each representative image. Increased deposition of ECM proteins and actin was observed in TM tissues of Dex-injected eyes compared to Veh-injected eyes (*n* = 4 or 6; TM, trabecular meshwork; CB, ciliary body)

Previously, we developed a mouse model of OHT by optimizing the formulation of Dex and its administration via periocular injection [40]. In this study, we examined whether Dex treatment leads to sustained IOP elevation that is sufficient to cause glaucomatous neurodegeneration. C57BL/6 J mice (3 months-old) were given bilateral periocular injections of either vehicle (Veh) or Dex once a week for 10 weeks and day and night IOPs were monitored. As shown in Fig. 1a, Dex led to sustained, pronounced and significant IOP elevation starting from the 1st week of injection. The mean IOP difference between Veh and Dex was ~ 4 mmHg during the daytime (Fig. 1a) and ~ 7 mmHg during the night-time (*S**I* 1). Outflow facility measurements revealed that Dex significantly (*p* = 0.027) reduced outflow facility by 37.8% after 4 weeks of injections (0.02 ± 0.009 μL/min/mmHg in Dex vs 0.0322 ± 0.001 μL/min/mmHg in Veh) (Fig. 1b). Importantly, Dex treatment did not alter body weight and both Veh and Dex-treated mice continued to gain weight at similar rates (*S**I* 2). Immunostaining for the major ECM proteins on iridocorneal angles of 10-week Veh and Dex-injected mice clearly showed increased ECM proteins and increased alpha smooth muscle actin expression in TM tissues of Dex-injected mice compared to Veh-injected mice (Fig. 1c, *S**I* 3). Slit-lamp images and H&E staining of 10-week Veh and Dex-injected eyes further confirmed the absence of any ocular abnormalities, and iridocorneal angles were open in both mice groups (*S**I* 4). Together, these data indicate that weekly injections of Dex lead to sustained IOP elevation, reduced AH outflow facility, and glaucomatous TM damage similar to human POAG.

### Dex-induced OHT leads to functional and structural loss of RGCs in mice

**Fig. 2:**
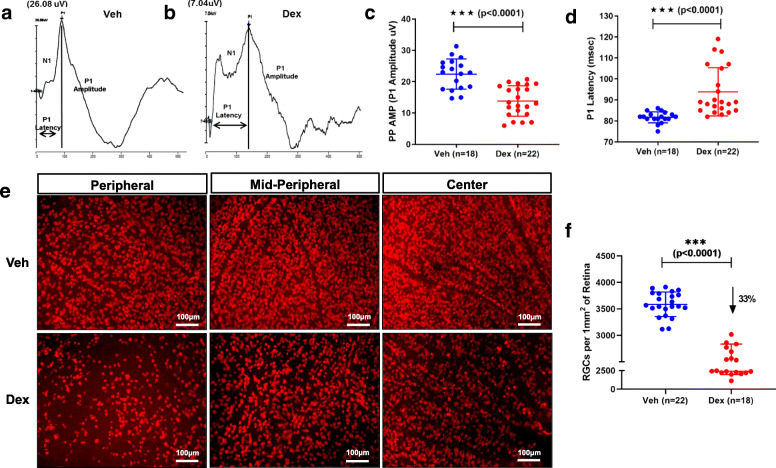
Dex-induced OHT leads to functional and structural loss of RGCs in mice. C57BL/6 J mice were periocularly injected with Veh or Dex for 10 weeks and RGC functional loss was examined by PERG. Representative wave graphs are shown for Veh (**a**) and Dex (**b**) injected mice. PERG amplitude (**c**) and latency (**d**) demonstrated a significant functional loss of RGCs in Dex-injected mice as evident from reduced amplitudes with increased latencies. Data are shown as mean ± SD (*n* = 18 in Veh and *n* = 22 in Dex, unpaired t-test, two tailed, ***p < 0.0001). **e** Representative images of immunostaining of whole mount retina with RGC specific marker, RBPMS in periphery, mid-periphery and center of retinas of 10 weeks post Veh or Dex-injected mice. A significant loss of RGCs (33% loss) is observed in Dex-injected mice (**f**). Data are shown as mean ± SD (n = 22 in Veh and n = 18 in Dex, unpaired t-test, two tailed, **p = 0.004, ***p < 0.0001)

To examine functional loss of RGCs, we measured pattern electroretinogram (PERG) amplitudes and latencies in 10-week Veh and Dex-injected mice. As shown in Fig. 2a-d, Dex-injected mice showed a significant (*p* < 0.0001) reduction in PERG amplitudes (13.81 ± 4.86 μV in Dex vs 22.42 ± 4.84 μV in Veh) and significantly delayed latency periods (93.86 ± 11.45 ms in Dex vs 81.72 ± 2.6 ms in Veh). Next, we examined whether Dex-induced OHT leads to RGC loss by whole mount retina staining with RGC specific marker, RBPMS. Total numbers of RGCs were analyzed from the whole mount retina in a masked and automated manner using Image J software in 10-week Veh and Dex-injected mice. Dex-injected mice showed significant RGC loss compared to Veh-injected mice (Fig. 2e&f). Overall, there was a ~ 33% RGC loss in Dex-injected mice compared to Veh-injected mice (Fig. 2f). Furthermore, H&E staining on cross sections of retinas demonstrated no gross morphological differences in retinal layers other than cell loss and thinning in the RGC layer in 10-week Veh and Dex-injected mice (*S**I* 5). These data indicate that Dex-induced OHT leads to both structural and functional loss of RGCs.

### Dex-induced OHT leads to optic nerve degeneration

**Fig. 3:**
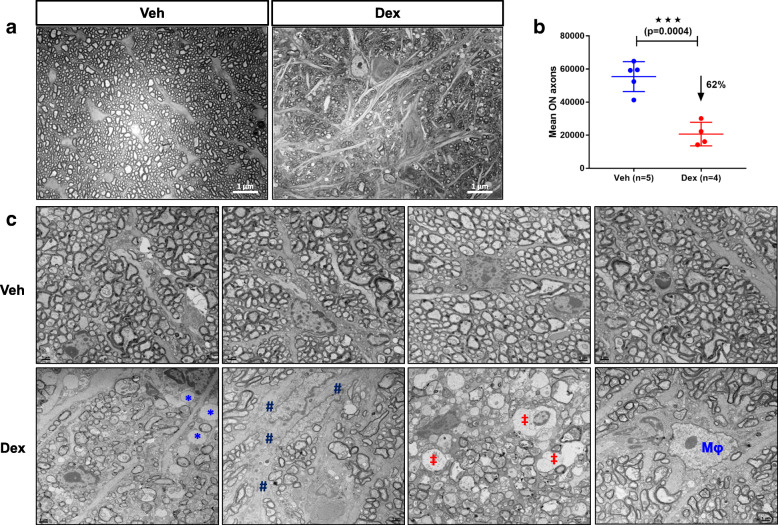
Dex-induced OHT leads to optic nerve axonal degeneration in mice. Optic nerves from 10-week Veh or Dex-injected mice were subjected to PPD staining (**a** & **b**) and TEM imaging (**C**) to examine optic nerve degeneration. **a** Representative images of PPD stained optic nerves show severe axonal degeneration along with gliosis and glial scar in 10 weeks Dex-injected mice compared to Veh-injected mice. **b** The mean axon counts in 10 weeks Dex-injected mice show a significant reduction in number of healthy axons compared to Veh-injected mice. Data are shown as mean ± SD (*n* = 5 in Veh and *n* = 4 in Dex, unpaired t-test, two-tailed, ***p = 0.0004). **c** TEM images confirm extensive glial scar and degenerated axons in Dex-injected mice compared to Veh-injected mice. In addition, we observed unmyelinated axons, vacuoles, infiltrating immune cells that were associated with optic nerve degeneration in Dex-injected mice compared to Veh-injected mice (*n* = 4). (* unmyelinated axons; **#** glial scar; **‡** vacuoles; **Mφ** infiltrating immune cells)

Optic nerve degeneration is the characteristic feature of human glaucoma and it is considered to be the critical pathological event of glaucomatous neurodegeneration that leads to vision loss [46, 47]. Therefore, we examined whether Dex-induced OHT causes optic nerve degeneration in mice using p-phenylenediamine (PPD) staining of optic nerves cross sections obtained from 10-week Veh or Dex-injected mice (Fig. 3a and *S**I* 6). Dex-injected mice demonstrated significant and pronounced optic nerve degeneration as evident from darkly stained axons, presence of vacuoles (*S**I* 7A), active gliosis and glial scar formation. Overall, 62% RGC axonal loss (*p* = 0.0004) was observed in 10-week Dex-injected mice (20,681 ± 7146) compared to Veh-injected mice (55,427 ± 9037) (Fig. 3b). We further determined ultra-structural changes associated with axonal degeneration using transmission electron microscopy (TEM) (Fig. 3c). Severe demyelination of optic nerve axons and extensive glial scar formation was observed in 10-week Dex-injected mice compared to Veh-injected mice. Dex-induced axonal lesions included hypo-myelinated and naked axons without any myelin sheath, axonal swelling, many residual empty vacuoles (*SI* 7A), increased mitochondrial accumulation per axon (*SI* 7B&C) and infiltration of mononuclear immune cells (i.e. macrophage-like cells) in the glial scar region.

### Reduction of elevated IOP prevents Dex-induced glaucomatous neurodegeneration

**Fig. 4:**
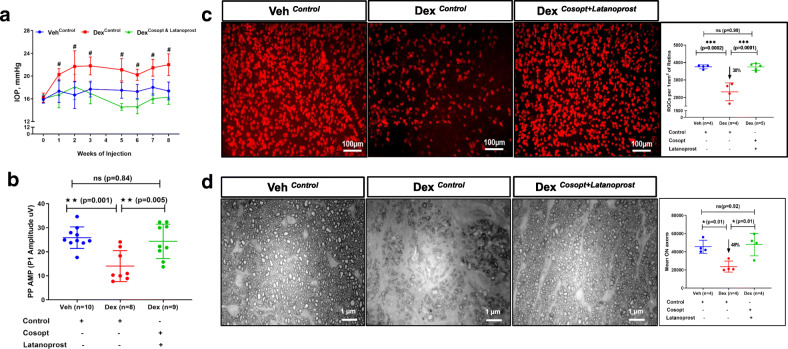
Dex-induced glaucomatous neurodegeneration is IOP dependent. Three-month old C57BL/6 J mice were injected bilaterally once a week for 8 weeks with either Veh or Dex, and given topical ocular eye drops of either control (water) or Cosopt (b.i.d) + Latanoprost (once a day) for 8 weeks. **a** Dex injected mice receiving control eye drops (Dex^*Control*^) show sustained and significant IOP elevation compared to Veh injected mice receiving control eye drops (Veh^*Control*^), IOP is significantly reduced in Dex-injected mice receiving Cosopt+Latanoprost eye drops (Dex^*Cosopt + Latanoprost*^) compared to Dex^*Control*^ mice. Data are shown as mean ± SD (*n* = 8 to 10 in each group, 2-WAY ANOVA with multiple comparison, #p < 0.0001). **b** Reduction of IOP prevents Dex-induced RGC functional loss. PERG was measured in mice treated with periocular injections of Veh or Dex along with or without topical ocular eye drops of Cosopt+Latanoprost. Dex^*Control*^ mice demonstrate significant reduction in PERG amplitudes compared to Veh^*Control*^ or Dex^*Cosopt + Latanoprost*^ mice. There is no difference in PERG amplitudes between Veh^*Control*^ and Dex^*Cosopt + Latanoprost*^ mice. Data are shown as mean ± SD (n = 8 to 10 in each group, One WAY ANOVA with multiple comparison). **c** Reduction of IOP prevents Dex-induced RGC structural loss. Representative images of whole mount retina immunostained with RBPMS and corresponding RGC counts are shown. There is a significant reduction in RGC numbers in Dex^*Control*^ mice compared to Veh^*Control*^ mice, whereas Dex^*Cosopt + Latanoprost*^ mice did not show RGC loss compared to Veh^*Control*^ mice. Data are shown as mean ± SD (*n* = 4 or 5, One WAY ANOVA with multiple comparison, ***p < 0.0001). **d** Reduction of IOP prevents Dex-induced optic nerve degeneration. PPD stained optic nerve axons were counted and total number of axons per optic nerve are represented in a dot plot. Dex^*Control*^ mice showed significant axonal degeneration compared to Veh^*Control*^ mice, and axonal degeneration was prevented in Dex^*Cosopt + Latanoprost*^ mice. Data are shown as mean ± SD (n = 4, One WAY ANOVA with multiple comparison, *p = 0.01)

We next determined whether glaucoma phenotypes observed in our model are truly IOP dependent or independent (i.e. any direct deleterious effects of Dex on RGCs). To address this, we reduced IOP in Dex-injected mice throughout the study period (starting from 1st day of injection till 8 weeks) by applying topical ocular eye drops of Cosopt (b.i.d) and Latanoprost (once daily) and compared glaucoma phenotypes with Veh and Dex-injected mice groups receiving control (water) eye drops. As shown in Fig. 4a, Dex^*Control*^ mice showed significant and sustained IOP elevation, whereas IOP in Dex^*Cosopt + Latanoprost*^ mice was reduced significantly starting from 1st week of injection. Dex^*Control*^ mice demonstrated prominent reduction in PERG amplitudes (14 ± 6.4 μV), whereas Dex^*Cosopt + Latanoprost*^ mice exhibited normal PERG amplitudes similar to Veh^*Control*^ mice (24.3 ± 7.1 μV in Dex^*Cosopt + Latanoprost*^ vs 25.8 ± 4.4 μV in Veh^*Control*^) (Fig. 4b). Consistent with PERG, whole-mount retina staining of RBPMS revealed significant loss of RGCs (38% loss) only in Dex^*Control*^ mice (but not in Dex^*Cosopt + Latanoprost*^ mice (Fig. 4c). Also, PPD staining of optic nerves demonstrated significant axonal degeneration in Dex^*Control*^ mice (~ 48% loss) compared to Veh^*Control*^ mice, while axonal degeneration was prevented completely in Dex^*Cosopt + Latanoprost*^ mice (Fig. 4d). In addition, infiltrated immune cells were observed in the degenerated optic nerves of Dex^*Control*^ mice, while reduction of IOP prevented immune cells infiltration in the optic nerves of Dex^*Cosopt + Latanoprost*^ mice similar to Veh^*Control*^ mice (***SI*** **8**). These data clearly indicate that reduction of IOP prevents Dex-induced glaucomatous neurodegeneration.

### Withdrawal of Dex reverses Dex induced OHT and its associated RGC loss

**Fig. 5:**
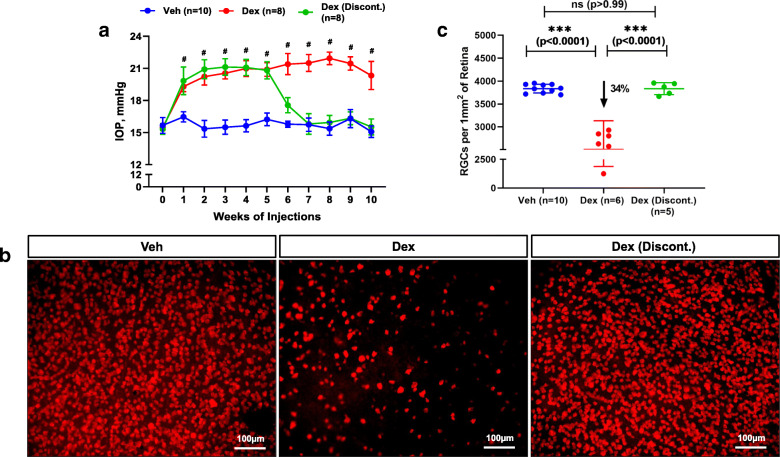
Withdrawal of Dex results in IOP reduction and prevents Dex-induced RGC loss. Three-month old C57BL/6 J mice were injected bilaterally once a week for 5-weeks with either Veh or Dex. Dex-treated mice were randomly divided in two groups. Dex treatment was withdrawn in one group while another group continued to receive Dex for additional 5 weeks. **a** Weekly IOP measurements revealed sustained and significant IOP elevation in Dex-injected mice compared to Veh-injected mice. Withdrawal of Dex injections caused significant reduction of elevated IOP in Dex discontinued group. Data are shown as mean ± SD (n = 8 to 10 in each group, 2-WAY ANOVA with multiple comparison, #p < 0.0001). **b** Representative RBPMS stained whole mount retinal images and **c** total RGC quantitation revealed a significant loss of RGCs in 10 weeks Dex-injected mice while no RGC loss was observed in Dex discontinued mice compared to Veh injected mice. Data are shown as mean ± SD (*n* = 10 in Veh, *n* = 6 in Dex and *n* = 5 in Dex discontinued group, One WAY ANOVA with multiple comparison, *p = 0.03, ***p < 0.0001)

We next determined whether Dex withdrawal prevents RGC loss. Three-month old C57BL/6 J mice were injected with either Veh or Dex via periocular injections once a week for 5 weeks. IOPs were monitored and IOP elevation in Dex-injected mice was ensured. After 5 weeks of injections, Dex-treated mice were randomly divided into two groups. Dex-treatment was discontinued in one group while other group continued to receive Dex for an additional 5 weeks. As shown in Fig. 5a**,** Dex induced IOP returned to baseline within a week upon withdrawal of Dex and no significant difference was observed in terms of IOP between Veh and Dex discontinued group of mice after withdrawal. However, mice that received Dex continuously for 10 weeks demonstrated sustained and significant IOP elevation. Mice that received Dex continuously for 10 weeks showed significant RGC loss compared to 10-week Veh injected mice (Fig. 5b). Overall, there was a ~ 34% RGC loss in 10-week Dex-injected mice compared to Veh-injected mice. However, Dex discontinued group of mice did not show RGC loss despite initial IOP elevation for 5 weeks (Fig. 5b&c) and the total number of RGCs remained similar between 10-week Veh injected and Dex discontinued mice (Fig. 5c). This data further indicated that Dex-induced RGC loss is IOP dependent and reduction of IOP by the withdrawal of Dex during the initial stages of disease onset can halt progression of glaucomatous neurodegeneration.

### Reactive astrocytes in the optic nerve head and retina of Dex-injected mice

**Fig. 6:**
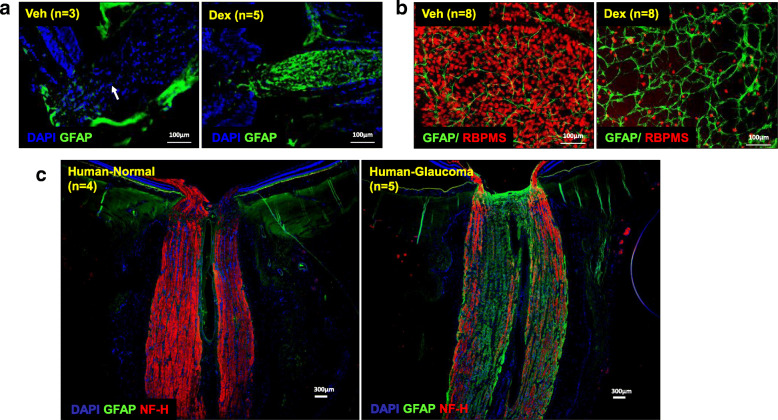
Sustained Dex-induced IOP elevation causes robust activation of astrocytes in ONH, ON and retina. Longitudinal sections of optic nerves (**a**) and whole mount retinas (**b**) were collected from 10 weeks post Veh or Dex-injected mice, and immunostained for reactive astrocytes with GFAP (green). **a** Hypertrophic reactive astrocytes with increased expression of GFAP were observed in ONH cross sections in 10 weeks Dex-injected mice (*n* = 5) compared to Veh-injected mice (*n* = 3). **b** Immunostaining of whole mount retinas with GFAP (green) and RBPMS (red) show relatively higher number reactive astrocytes and decreased RGCs in 10 weeks Dex-injected mice compared to Veh-injected mice (n = 8). **c** For comparisons, longitudinal sections of aged-matched human normal and glaucomatous optic nerves were immunostained with antibodies specific for GFAP and neurofilament-heavy chain (NF-H) (red). Loss of NF-H with increased GFAP expressing reactive astrocytes are observed throughout the length of human glaucomatous optic nerve compared to aged-matched normal human optic nerve. In addition, there is a robust activation of astrocytes in human glaucomatous ONH (n = 5) compared to normal ONH (n = 4)

The abnormal activity of reactive astrocytes is known to be associated with glaucomatous neurodegeneration [48–52]. We therefore determined whether Dex-induced glaucomatous neurodegeneration is associated with astrocyte activation. Optic nerves and retinas from 10-week Veh or Dex-injected mice were stained with glial fibrillary acidic protein (GFAP) antibody. A strong GFAP staining was observed in the ONH of Dex-injected mice compared to Veh-injected mice (Fig. 6a**,** *S**I* 9A). In addition, we observed relatively higher GFAP in the inner retina of Dex-injected mice (Fig. 6b). Similar to these findings, a prominent GFAP staining was observed in the ONH of human glaucomatous optic nerve compared to age-matched normal optic nerves (Fig. 6c**,** *S**I* 9B). These data clearly indicate that Dex-induced glaucomatous neurodegeneration is associated with reactive astrocytes, similar to human glaucoma.

### Dex-induced OHT leads to anterograde axonal transport deficits in the ONH

**Fig. 7:**
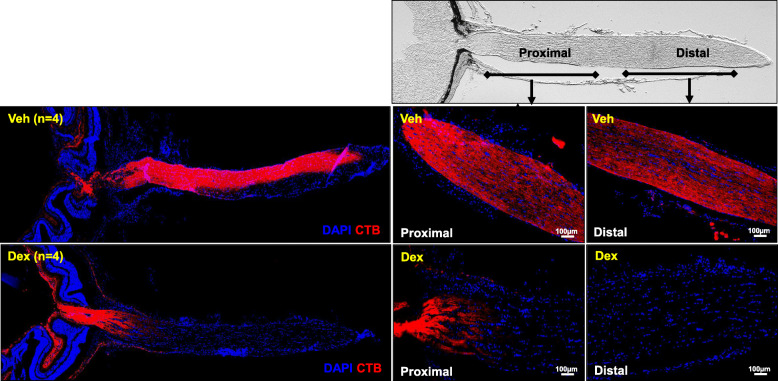
Dex-induced OHT leads to axonal transport deficits in mice. C57BL/6 J mice treated with Veh and Dex for 10 weeks and injected intravitreally with red fluorescently tagged CTB dye (Alexa fluor 555). The proximal (close to ONH) and distal (close to optic chiasm) regions of optic nerve are marked in DIC image of optic nerve on the upper panel. Veh-injected mice showed an uninterrupted axoplasmic anterograde transportation of CTB along the entire length of optic nerve including both proximal and distal regions. However, CTB transport was blocked completely at ONH and no CTB was detected in distal region of optic nerve (n = 4)

Anterograde transport blockage at the ONH is associated with glaucomatous neurodegeneration [53, 54]. We therefore investigated whether the axonal transport deficits are associated with Dex-induced neurodegeneration using fluorescently tagged cholera toxin (CT) B dye. C57BL/6 J mice were injected with Veh or Dex for 10 weeks, and CTB was injected intravitreally. 48 h after injections, CTB transport was examined in the retina and ONH (Fig. 7). Veh-injected mice demonstrated a continuous and uninterrupted transport of CTB along the entire length of optic nerve. However, in Dex-injected mice, most of CTB accumulated in the proximal region of ONH and no dye was transported to the distal region of optic nerve (Fig. 7**)**. These results indicate that Dex-induced OHT leads to blockage of anterograde axonal transport at the ONH.

### Infiltration of F4/80^+^ activated macrophage-like cells in the optic nerve of Dex-injected mice and human POAG donor tissues

**Fig. 8:**
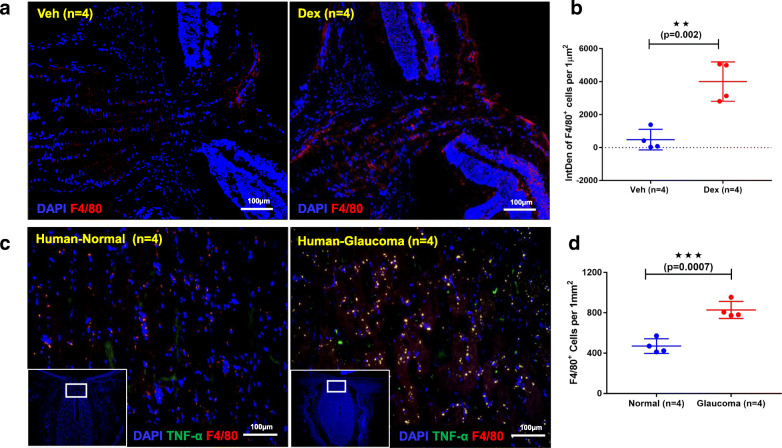
Glaucomatous neurodegeneration is associated with activation of F4/80 positive macrophage-like cells. **a**&**b** 10 weeks Veh and Dex-injected eyes are enucleated, sectioned through ONH and analyzed for F4/80 positive cells by immunostaining. The corresponding images from 10 weeks post Dex-injected eyes showed an increased number of F4/80 positive macrophage-like cells at ONH compared to 10 weeks Veh injected eyes (n = 4). **c**&**d** For comparisons, a similar trend is observed in the human glaucomatous ONH, where the number of F4/80 positive macrophage-like cells expressing TNF-α (green) is significantly higher in glaucomatous ONH compared to age matched normal ONH (n = 4 in normal and n = 4 in glaucoma). Data are shown as mean ± SD (unpaired t-test, two tailed, **p = 0.002, ***p = 0.0007)

TEM analysis of optic nerve cross sections from Dex-injected mice revealed the presence of infiltrating immune cells associated with optic nerve degeneration (Fig. 3c). Therefore, we confirmed the nature of infiltrating immune cells by F4/80 antibody staining on sections of optic nerves from 10-week Veh and Dex-injected mice (Fig. 8a&b). Numerous infiltrating F4/80^+^ activated macrophage-like cells were observed in the ONH of Dex-injected mice. We further examined whether similar F4/80^+^ activated macrophage-like cells are also present in ONH tissues from human POAG donors. Age-matched normal (*n* = 4) and glaucomatous (n = 4) ONH sections were stained with antibodies for F4/80 and TNF-α. Striking infiltration of F4/80^+^ activated macrophage-like cells producing proinflammatory cytokine TNF-α were observed in human glaucomatous ONH compared to age-matched normal donor tissues (Fig. 8c&d). These data indicate that Dex-induced axonal degeneration is associated with infiltrating immune cells similar to human POAG.

### Optic nerve degeneration precedes structural and functional loss of RGCs in mouse model of Dex-induced glaucoma

**Fig. 9:**
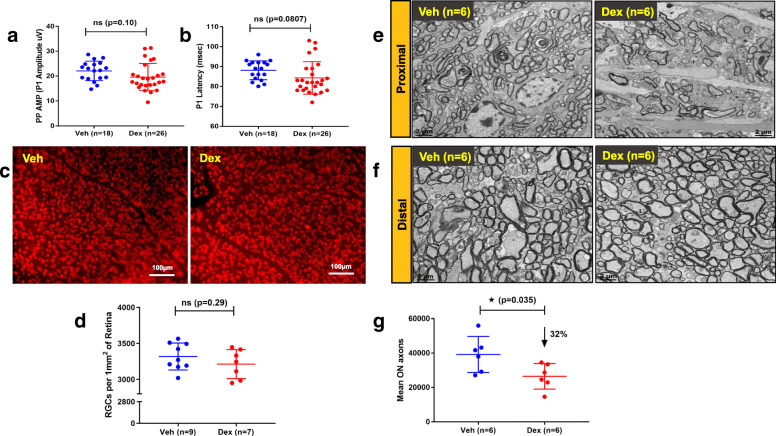
Optic nerve degeneration precedes structural and functional loss of RGCs. Three-month old C57BL/6 J mice were injected bilaterally once a week for 5 weeks with Veh or Dex. The structural and functional loss of RGCs was analyzed by PERG and immunostaining (**a**-**d**). Optic nerve degeneration was examined via TEM and PPD staining (**e**-**g**). Dot graphs show PERG amplitudes (**a**) and latency (**b**) demonstrating no significant loss of RGC function in Dex-injected mice after 5 weeks of treatment. Data are shown as mean ± SD (*n* = 18 in Veh and *n* = 26 in Dex, unpaired t-test, ns = not statistically significant, *p* > 0.05). In consistence with PERG data, the flat mount retinal images stained with RBPMS (**c**) and the corresponding dot plots (**d**) showed no significant RGC loss between the 5 weeks post Veh and Dex injected mice (*n* = 9 in Veh and *n* = 7 in Dex). The data are shown as mean ± SD (unpaired t-test, two tailed, ns = not statistically significant, p > 0.05). The representative TEM images (**e**&**f**) show axonal degeneration and glial scar formation in proximal region (close to ONH) (**e**) and not at distal ON region of 5 weeks post Dex injected compared to Veh injected mice (n = 6) (**f**). The mean optic nerve axonal counts at the proximal region of optic nerves show a significant axonal loss in 5 weeks Dex-treated mice compared to control mice (**g**). The data are shown as mean ± SD (n = 6, unpaired t-test, two tailed, *p* = 0.035)

We further sought to understand the early events of glaucomatous axonal degeneration in our mouse model. Since we observed RGC cell death and optic nerve degeneration at 8 and 10 weeks of Dex treatment, we evaluated glaucomatous neurodegeneration at an early time period of Dex treatment (5 weeks). We observed no significant differences in PERG amplitudes (22.07 ± 3.95uV in Veh and 19.59 ± 5.45uV in Dex) or latency periods (88.11 ± 4.75 msec in Veh and 84.27 ± 8.1 msec in Dex) in 5-week Veh or Dex-injected mice (Fig. 9a&b and *SI* 10). Consistent with these data, we also observed no significant loss of RGCs in 5-week Veh or Dex-injected mice (Fig. 9c). Enumerating RGCs from whole mount retina revealed that the total number of surviving RGCs were similar in both Veh and Dex-injected mice (Fig. 9d). These data established that Dex-induced OHT for 5 weeks does not lead to functional or structural loss of RGCs soma. Next, we examined axonal degeneration of optic nerves after 5 weeks of Dex treatment. Previous studies have suggested that axonal degeneration can be compartmentalized and both dying back and Wallerian degeneration were proposed as mechanisms of distal axonal degeneration in mouse models of glaucoma [55, 56]. We therefore examined the axonal degeneration pattern in our model at both proximal (close to ONH) and distal (close to optic chiasm) regions of optic nerves by TEM analysis and PPD staining. Despite no RGC loss, TEM imaging showed significant axonal degeneration in 5-week Dex-injected mice as evident from loss of healthy myelinated axons as well as the presence of astrocyte processes at the proximal region of optic nerves (Fig. 9e). However, no axonal injury was evident at the distal region of optic nerves in 5-week Dex-treated mice. Also, the morphology of axons at distal region of optic nerves of Dex-injected mice was undistinguishable from Veh-injected mice (Fig. 9f). Immunostaining data further revealed that the proximal axonal injury in 5 weeks Dex-injected mice was associated with significant increase in reactivation of astrocytes and moderate increase in immune cell infiltration at ONH (*S**I* 11). Since we observed axonal injury at the proximal region of Dex-injected optic nerves, we further quantified healthy myelinated axons using PPD staining. Approximately 32% loss of optic nerve axon was observed in 5-week Dex-injected mice compared to Veh-injected mice (Fig. 9g). Contrary to this, PPD staining on 10-week Dex-injected optic nerves showed similar degenerative patterns at both proximal and distal ends of optic nerves (*S**I* 12). These data indicate that Dex-induced OHT initiates axonal degeneration at the proximal region of optic nerves. Moreover, Dex-induced optic nerve degeneration precedes structural and functional loss of RGC soma.

### Anterograde axonal transport mechanisms persist during initial stages of axonal degeneration

**Fig. 10:**
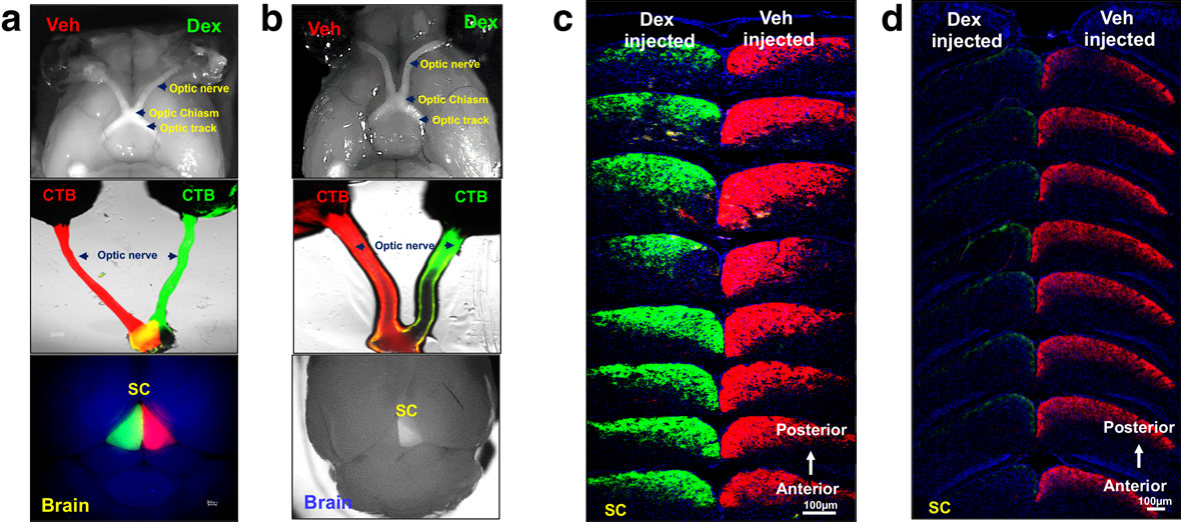
Anterograde transport persists during initial stages of optic nerve degeneration. Three-month old C57BL/6 J mice were injected unilaterally with Veh and Dex once a week for 5 or 8 weeks and axonal anterograde transport deficits were tracked through entire visual pathway including optic nerve and superior colliculus (SC) using fluorescently tagged CTB. CTB Alexa Fluor 555 (Red) and CTB Alexa Fluor 488 (Green) were used to tract transport deficits in Veh and Dex-injected eyes respectively. **a** Representative DIC images connecting eyes to the brain from 5 weeks injected mice show an active transportation of CTB in both Veh and Dex-injected eyes. **b** Representative DIC images show accumulation of CTB (green fluorescence) at the ONH in 8 weeks Dex-injected eyes compared to contralateral Veh-injected eyes (CTB-red fluorescence). **c** Serial frontal sections of brain confirm a loss of axonal transport of CTB with mild deficits in the posterior SC regions in 5 weeks Dex-injected eyes compared to contralateral Veh-injected eyes (n = 5). **d** Serial frontal sections of brain confirm the axonal transport deficits of CTB in SC regions corresponding to 8 weeks Dex-injected eyes compared to contralateral Veh injected eyes indicating complete axonal anterograde transport deficits during later stages of axonal damage (n = 5)

Since we observed mild optic nerve degeneration without any functional or structural loss of RGCs at 5 weeks of Dex treatment, we explored whether the anterograde transport mechanism is impaired in the optic nerves of 5-week Dex-injected mice. C57BL/6 J mice were periocularly injected with Veh in one eye while the contralateral eye was injected with Dex for 5 or 8 weeks and IOPs were monitored weekly to ensure OHT. To analyze axonal anterograde transport, Veh-injected eyes were intravitreally injected with red fluorescently tagged CTB dye while the contralateral Dex-injected eyes were intravitreally injected with green fluorescently tagged CTB dye at 5 and 8 weeks (Fig. 10 a-d). 48-h after injections, anterograde transport of CTB was tracked through the entire optic nerve as well as visual centers of brain including the superior colliculus (SC). Gross examination of CTB at 5 weeks of treatment revealed similar levels of CTB transport along the entire optic nerve and into the SC in both Veh and Dex-injected eyes (Fig. 10a). However, axonal transport of CTB was completely blocked at the ONH in 8-week Dex-injected eyes compared to Veh-injected contralateral eyes (Fig. 10b). Consistent with this, no CTB transport was detected at the SC in 8 weeks Dex-treated eyes. We further examined CTB transport to the visual centers using serial sections of the SC at 5 (Fig. 10c) and 8 (Fig. 10d) weeks of treatment. When we analyzed CTB transport in entire SC using serial frontal sections, we observed mild transport deficiency in posterior regions of SC after 5 weeks of Dex treatment (Fig. 10c). In addition, no CTB was detected in any SC layers after 8 weeks of Dex treatment (Fig. 10c). These data indicate that majority of anterograde transport persists to visual centers during initial stages of axonal degeneration and the degree of anterograde transport deficits are dependent on severity of axonal degeneration.

### Partial axonal transport deficits at the ONH are associated with early stages of optic nerve degeneration in Dex-treated mice

**Fig. 11:**
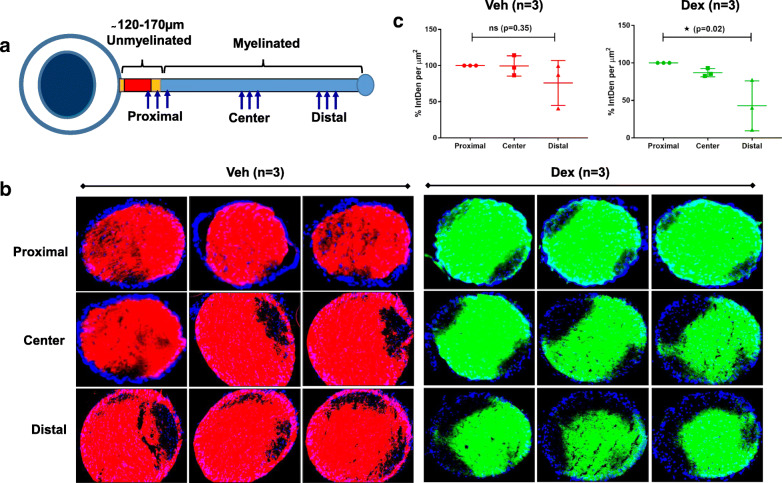
Partial axonal transport deficits at the ONH is associated with early stages of optic nerve degeneration in Dex-treated mice. Three months old C57BL/6 J mice were injected unilaterally with Veh and Dex once a week for 5 weeks and axonal anterograde transport was tracked along the entire length of optic nerve using fluorescently tagged CTB. CTB Alexa Fluor 555 (Red) and CTB Alexa Fluor 488 (Green) were used to tract transport deficits in Veh and Dex-injected eyes respectively *n* = 3. **a** Graphical representation of serial cross sections of optic nerve at the proximal (close to glial lamina), center and distal (close to optic chiasma) regions. **b** Serial cross sections of optic nerve images show partial accumulation of CTB (green fluorescence) at proximal ON region in 5 weeks Dex injected eyes compared to contralateral Veh-injected eyes (CTB-red fluorescence). **c** Fluorescent intensities of CTB from proximal to distal ON in 5 weeks of Veh and Dex injected mice was analyzed by Image J and shown graphically. For each optic nerve, an average of fluorescent intensities of 3 images from each region were taken into the consideration. The data are shown as mean ± SD (n = 3, One WAY ANOVA with multiple comparison; ns = not significant, *p = 0.02)

Since we detected partial deficits in the transport of CTB to the SC at 5 weeks of Dex treatment, we further explored whether these transport deficits at the SC are the result of degenerative processes at the SC or ONH. As shown in Fig. 10a, gross analysis of CTB transport appears to be normal in both Veh and Dex-treated eyes. We further performed serial cross sections at proximal, center and distal regions of optic nerve at 5 weeks treatment (Fig. 11a), and fluorescent intensities were analyzed and measured in different regions (Fig. 11b&c). While most of the CTB (red fluorescence) was transported uniformly throughout optic nerves in Veh-treated eyes, higher fluorescent intensities of CTB (green fluorescence) were observed near the proximal regions compared to distal regions of optic nerves in Dex-injected eyes. We observed a 25% reduction in fluorescent intensities of CTB from proximal to distal ends of the optic nerve in Veh-injected eyes whereas ~ 50% reduction was observed in Dex-injected eyes (Fig. 11**c**). These data indicate that there is a partial blockage of CTB transport in Dex-treated optic nerve progressing from proximal to distal. These data further suggest that partial axonal transport deficits observed in SC region of Dex-treated mice are likely due to blockage of transport at the ONH.

## Discussion

GC-induced OHT and its associated iatrogenic open-angle glaucoma share many features with POAG. Therefore, several laboratories have attempted to develop mouse models of GC-induced glaucoma using either systemic or topical ocular administration of GCs. However, most of these studies were limited to study GC-induced OHT most likely due to insufficient IOP elevation to cause glaucomatous neurodegeneration. We recently developed a mouse model of GC-induced OHT via periocular injections of Dex, which led to pronounced IOP elevation. However, we did not determine whether Dex-induced IOP elevation was sustained and sufficient to develop glaucomatous neurodegeneration similar to human POAG. In this study, we demonstrate that weekly injections of Dex lead to sustained and pronounced IOP elevation, which is associated with reduced AH outflow and dysfunction of TM similar to POAG. Our studies clearly show that Dex-induced OHT leads to robust and pronounced glaucomatous neurodegeneration, including functional and structural loss of RGCs and optic nerve degeneration. Optic nerve axonal degeneration is also associated with reactive astrocytes, transport deficits and infiltration of macrophage-like cells in the ONH. Using this model, we evaluated early events of glaucomatous neurodegeneration, which is crucial to understand the etiology of glaucoma and to develop novel therapeutic strategies to halt disease progression. Here, we demonstrate that IOP-induced axonal damage occurs at the ONH and rapidly progresses toward the distal ends of the optic nerve. Optic nerve degeneration precedes structural and functional loss of RGCs in response to Dex-induced OHT. By analyzing axonal transport mechanisms at 3 different crucial time points (i.e. 5, 8 and 10 weeks) of sustained IOP elevation, we present evidence that the majority of active anterograde axonal transport persists during the early stage of optic nerve degeneration (32% axonal loss at 5 weeks), while complete transport deficits only occur at later stages of axonal damage (more than 50% axonal loss at 8 and 10 weeks of treatment).

Although there are several mouse models to study pathophysiology of glaucomatous neurodegeneration, most of these models do not faithfully replicate human POAG. We have previously developed a transgenic mouse model of myocilin glaucoma and demonstrated that transgenic mice exhibit glaucoma phenotypes similar to myocilin patients [41]. However, glaucomatous neurodegeneration in this model is slow and requires aging of mice. Therefore, there is critical need to develop inducible mouse models of POAG that develop robust glaucomatous neurodegeneration. Our Dex-induced glaucoma mouse model demonstrates striking similarities with human POAG. These features include open iridocorneal angle, IOP elevation caused by reduced outflow facility due to TM dysfunction, and biochemical and morphological changes in the TM similar to POAG. In addition, Dex leads to IOP dependent progressive optic nerve degeneration and loss of RGCs. Specifically, lowering of IOP prevents Dex-induced glaucomatous neurodegeneration. Studies on humans demonstrated that discontinuation of GC therapy normalizes the IOP within 1 to 4 weeks [26, 57] by restoring the outflow facility and may halt the disease progression. Previous studies from our group also observed withdrawal of Dex lowers down IOP within 1 or 2 weeks in mouse models of OHT depending on route of administration [40]. Consistent with these studies, we also observed that withdrawal of Dex normalized the IOP within 7 to 10 days and halted the progression of RGC loss. Together, these studies suggest that Dex-induced glaucoma is truly dependent on IOP.

**Fig. 12:**
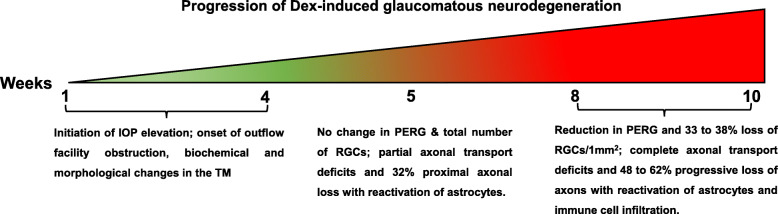
The schematic diagram showing the timeline of the progression of glaucomatous neurodegeneration in mouse model of Dex-induced OHT. Dex treatment leads to significant IOP elevation starting from 1 week of injection, which is associated with reduced outflow facility. These changes are associated with ECM accumulation and increased cytoskeleton proteins in the TM. About 32% ON axonal loss and activation of astrocytes in ONH was observed at 5 weeks of Dex treatment. At this stage, we did not observe functional or structural loss of RGCs. Most of the CTB transport was active at this stage. At 8 and 10 weeks of treatment, there was 48 and 62% loss of optic nerve axons respectively. At this stage, significant structural (30–40%) and functional loss of RGCs was observed. We also observed complete blockage of axoplasmic transport along with activation of astrocytes and infiltration of immune cells

Despite several attempts to develop mouse model of Steroid-induced glaucoma, this is the only mouse model of Steroid-induced ocular hypertension that develops robust and reproducible glaucomatous neurodegeneration. Our Dex-induced glaucoma model offers several advantages and exhibits unique features of glaucomatous neurodegeneration that were not observed previously in other inducible mouse models of glaucoma. First, unlike current models, IOP elevation in our Dex mouse model is largely due to TM dysfunction, and it is associated with reduced outflow facility similar to POAG. Second, Dex-induced glaucomatous neurodegeneration is truly IOP-dependent and demonstrates reproducible phenotypes. More than 90% of eyes treated with Dex develop OHT and glaucomatous neurodegeneration. Third, the exact timeline of glaucomatous neurodegeneration can easily be tracked. IOP elevation starting from the 1st week of injections, optic nerve degeneration can be observed at 5 weeks of treatment, which is followed by structural and functional loss of RGCs and complete blockage of axonal transport at 8 weeks (Fig. 12). These features make our Dex-induced glaucoma model ideal to study pathophysiology of glaucomatous damage to TM tissue, RGC loss, and axonal degeneration. Furthermore, this model can be effectively utilized to screen new treatment strategies targeted to reduce IOP or to develop novel neuroprotective agents.

GCs are known to have strong anti-inflammatory and immune suppressive properties, and are often used for the treatment of traumatic optic neuropathy and optic neuritis to inhibit inflammation [58]. In our model, it is intriguing to note the presence of reactive astrocytes and F4/80^+^ immune cells associated with optic nerve degeneration. It is likely that Dex effects are localized to the TM region and there is no direct effect of Dex on the optic nerve and RGCs. Lowering of IOP rescued Dex-induced glaucomatous neurodegeneration further supporting our hypothesis that Dex injected via the periocular route has no direct effect on RGCs or ONH cells.

Our study clearly demonstrates that initial insult to neurons occurs in the glial lamina region of the optic nerve similar to that observed in human and primate models of glaucoma [49], and it is accompanied by numerous cellular responses including activation of astrocytes and infiltration of immune cells [50, 59–63]. Also, optic nerve degeneration precedes structural and functional loss of RGCs in our Dex mouse model of glaucoma. Consistent with our findings, some studies using the DBA/2 J mouse model also reported that axonal dysfunction and degeneration occurs before loss of RGCs [55, 64]. Our experimental data based on TEM imaging from 5- and 10-week post Dex-induced OHT mice further suggest that optic nerve degeneration occurs distal to the RGCs soma initiating at the ONH and rapidly progressing towards the distal region of optic nerve following Wallerian type axonal degeneration. Axonal degeneration is associated with the presence of reactive astrocytes, glial scar and infiltrating macrophage-like cells. Similarly, activated astrocytes were also observed in the ONH of human POAG tissues.

Our Dex-model allows the study of key early pathological events of glaucomatous neurodegeneration using different time periods of OHT (5, 8 and 10 weeks) and we discovered several new mechanistic insights that were not previously described. The pattern of axonal degeneration is quite different than reported in previous studies. These differences include presence of hypomyelinated and naked optic nerve axons with the presence of vacuoles. It is possible that these features of early stages of axonal degeneration are unique to this Dex model. Anterograde axonal transport deficits have been shown to precede retrograde transport deficits [65] and studies by Crish et al. 2010 demonstrated that distal transport deficits at SC are prodegenerative in DBA/2 J mice [56]. However, it is not completely understood whether anterograde axonal transport deficits precede or proceed optic nerve degeneration or loss of RGCs in mouse models of POAG. Utilizing precise timeframes of neurodegeneration, our studies clearly demonstrate that most axonal transport persists through the optic nerve to visual centers of the brain despite initial optic nerve degeneration (~ 32% loss of axons at 5 weeks). A complete blockage of axonal transport was only observed in optic nerve after 8 weeks of OHT (~ 48% loss of axons). Although gross analysis did not reveal any discernable changes in the transport of CTB to the SC at 5 weeks of OHT, it is interesting to note a partial block of transport of CTB in posterior layers of SC when serial sectioning of the SC was performed. In contrast, a complete blockage of CTB was observed at the ONH and no CTB was transported into the SC in 8-week Dex-treated mice. CTB transport deficits to the SC at 5 weeks of Dex treatment could be due to degenerative processes in the SC or ONH. To further clarify this, we performed serial cross sections of optic nerves, which revealed that partial transport deficits in the ONH of 5 weeks Dex-treated eyes. Although there was relative loss of transport at 5 weeks, the fact that most of the CTB was transported to the SC suggests that the majority of anterograde transport is active during initial stages of optic nerve degeneration. These data also indicate that optic nerve degeneration and anterograde transport deficits precede RGC soma loss. The presence of most of active axonal transport despite axonal injury in 5-week Dex-injected mice suggests existence of intact axonal cytoskeletal machinery or transport compensation through the rest of healthy axons at initial stages of degeneration. Since axonal transport is a cell survival process, the functional integrity of active axonal transport in 5-week Dex-injected mice may support RGCs soma survival, which can explain no structural or functional loss of RGC soma at this stage. This also opens up a potential window for regeneration strategies to support dying axons along with effective reduction of IOP to prevent further neurodegeneration. Our mouse model provides well-defined molecular events that lead from IOP elevation to axonal degeneration.

Contrary to the studies in DBA/2 J, which proposed both dying back and Wallerian degeneration as mechanisms of axon degeneration [55, 56], axonal degeneration in our mouse model closely resembles a Wallerian degeneration-like pattern. This is evident from focally swollen and damaged axons as well as the presence of vacuoles. When we examined optic nerve in the proximal versus distal regions of the ONH of the same eye, we observed degeneration only in proximal optic nerve at initial stages of neurodegeneration (5-weeks). However, optic nerve degeneration was observed in both proximal and distal regions of optic nerve at a later stage of degeneration (10 weeks), further supporting that degeneration initiates at the ONH region and progresses toward the distal end of optic nerve at later stages of degeneration. Also, we observed increased accumulation of mitochondria in this mouse model indicating axonal vulnerability to mitochondrial dysfunction or altered mitochondrial biogenesis, which may further affect neuronal functions including energy demands, metabolic balance, apoptotic signaling and intracellular calcium homeostasis. Axonal cytoskeletal distortion and mitochondrial dysfunction further leads to complete deficits in axonal transportation. In addition, hypomyelinated and naked axons with severe demyelination are observed in our mouse model, which may halt the neurotransmission of action potentials. Importantly, we noted reactive astrocytes and infiltrating F4/80^+^ activated macrophage-like cells during the axonal degeneration process in both human glaucoma and in our mouse model of glaucoma. Similar to our findings, monocyte infiltration into the ONH and its role in RGC damage has been reported in DBA/2 J model [62, 63]. Reactive astrocytes and F4/80^+^ activated macrophage-like cells observed in our mouse model are detrimental for axonal regeneration as they modulate complement activation, endothelin signaling and secretory proinflammatory cytokines such as TNF-α [62, 66].

As a consequence of severe axonal degeneration, we observed significant RGC loss with reduced PERG amplitudes with increased latency periods in our mouse model. The structural integrity of the remaining retinal layers are not affected by the IOP induced damage. In our mouse model, the estimated loss of RGCs (33%) is milder when compared to axonal loss (62%). Although these data suggest that RGC axonal loss precedes structural and functional loss of soma, it is possible that there are significant changes in dendritic structures in certain types of RGCs. Previous studies have clearly shown significant dendritic changes in the RGCs after a brief exposure of OHT [67, 68]. Since we did not detect any significant reduction of PERG at 5-weeks of treatment, it is possible that these dendritic changes have minimum effect on RGC function. Also, the observed loss of RGCs in retinal whole mount of our mouse model is not uniform where the loss of RGCs is most severe in the periphery compared to mid-periphery and center. In this study, we observed the loss of RGCs is restricted to two quadrants in a few of whole mount retinas from Dex-injected mice. A similar RGC loss pattern is reported by Jakobs et al., using the DBA/2 J mouse model of inherited chronic glaucoma [69]. A previous study by Zode et al., reported 16% of RGC loss after 20-weeks of daily administration of topical eye drops of Dex [38]. However, ~ 33% loss of RGCs was observed at 10 weeks of Dex-treatment in our current model. RGC loss was analyzed at 3 different time periods. We observed no loss of RGCs at 5 weeks, 38% loss of RGCs at 8 weeks (eye drop group), and 33% loss of RGCs at 10 weeks of Dex treatment. RGC loss at 8-weeks of Dex treatment was higher than 10 weeks. This is likely due chronic handling of mice for daily eye drops and low sample size. We also observed less weight gain in mice treated with eye drops compared untreated mice. Nonetheless, it is possible that Dex-induced RGC loss is variable in this model. It is interesting to note that Dex-induced optic nerve degeneration consistently declined with time period of treatment. We observed about 32% loss of axons at 5 weeks, 48% loss at 8 weeks and 62% loss at 10 weeks of Dex treatment (Fig. 12).

## Conclusions

We developed a simple and highly reproducible mouse model of glaucoma that faithfully mimics human glaucoma including IOP elevation induced by TM dysfunction and reduction of AH outflow, IOP-dependent glaucomatous neurodegeneration, axonal degeneration associated with robust activation of astrocytes, demyelination and immune cell infiltration and RGC loss. Utilizing this model, we define molecular events of glaucomatous neurodegeneration that occur as a result of chronic IOP elevation. Strikingly, we show that optic nerve degeneration preceded RGC structural and functional loss. Also, axonal transport mechanisms persist during initial stages of optic nerve degeneration. These findings provide a potential therapeutic window to target early changes of axonal degeneration for the treatment of glaucoma.

## Supplementary information

**Additional file 1: Fig. S1.** Dex-induced IOP elevation is higher during the night-time. C57BL/6 J mice were periocularly injected with Veh or Dex in both eyes, and IOPs were monitored weekly in the dark during the night time under isoflurane anesthetic conditions. Dex injections lead to sustained and significant IOP elevation and the observed mean difference of Dex-induced IOP is more pronounced at night compared to daytime IOP. Data are shown as mean ± SD (*n* = 8 in each group, 2-WAY ANOVA with multiple comparison, ***p = 0.0002, #*p* < 0.0001). **Fig. S2.** Effect of periocular administration of Dex on body weight in mice. 3-months old C57BL/6 J mice were periocularly injected with Veh or Dex weekly for 10 weeks, and body weight was monitored before and every week after injections. No significant difference in body weight between Veh and Dex-injected mice was observed. Both Veh and Dex-treated mice continued to gain weight as expected. Data are shown as mean ± SD (*n* = 5 in each group, 2-WAY ANOVA with multiple comparison). **Fig. S3.** Dex induced ECM proteins deposition and cytoskeleton changes in mouse TM. Densitometry analysis confirmed significantly increased deposition of major ECM proteins FN, ColI and laminin and also increased cytoskeleton markers including alpha smooth muscle (SMA) and phalloidin in the TM tissues of 10 weeks Dex injected mice compared to Veh injected mice. Data are shown as mean ± SD (*n* = 4 or 6 in each group, unpaired t-test, two tailed, **p = 0.002, #p < 0.0001). **Fig. S4** Effect of periocular Dex administration on ocular tissues. Slit lamp imaging and H&E staining was performed in 10 weeks Veh or Dex-injected mice. Slit lamp images (left panel) demonstrate no ocular abnormalities. H & E staining reveals no changes in gross morphology of iridocorneal angles as well as no other ocular structural abnormalities in Veh or Dex-injected eyes (*n* = 6). **Fig. S5.** Effect of Dex-induced OHT on retinal layers. **A**) Representative images of H&E staining on retinas of 10 weeks Veh and Dex-injected mice. **B**) IPL layer thickness was measured and represented graphically. Except for the cell loss in the RGC layer, there were no gross morphological differences in other retinal layers including IPL between Veh and Dex-injected eyes (*n* = 3 in each group, ns = not significant, *p* = 0.5). **Fig. S6.** Optic nerve axonal degeneration in Veh and Dex-injected mice. PPD staining was performed on cross sections of optic nerves collected from 10 weeks Veh or Dex-injected mice. Optic nerves from Dex-injected mice show severe axonal degeneration with extensive glial scar formation and reduced number of healthy axons with severe vacuolization patterns compared to optic nerves from Veh injected mice (n = 6 in each group). **Fig. S7**. Presence of vacuoles and accumulation of mitochondria in association with Dex induced axonal degeneration. C57BL/6 J mice were periocularly injected with Veh or Dex for 10 weeks, and (**A**) vacuoles were counted from PPD stained cross sections of ON. (**B&C**) Mitochondrial accumulation was evaluated by immunostaining with Cox4 and Cox4 florescent intensity was measured and shown graphically. The representative dot plots demonstrated significantly increased number of vacuoles as well as mitochondrial accumulation in Dex-treated mice compared to Veh-treated mice (**A&C**). Data are shown as mean ± SD (*n* = 3–5 in each group, unpaired t-test, two tailed, *p = 0.03, ***p = 0.0003). **Fig. S8.** Reduction of elevated IOP prevents immune cells infiltration in mouse optic nerve. Enlarged images of PPD stained cross sections of optic nerve from Dex^*Control*^ mice showed presence of infiltrated immune cells in the degenerated optic nerves. Reduction of IOP prevents immune cells infiltration in optic nerve of Dex^*Cosopt + Latanoprost*^ mice. (n = 3, the arrow heads pointed toward infiltrated immune cells). **Fig. S9.** Presence of reactive astrocytes in the glaucomatous ONH. A representative dot plots showed significant increase in activation of astrocytes at ONH region of 10 weeks Dex injected mice (**A**) and in human glaucomatous ONH region (**B**). Data are shown as mean ± SD (unpaired t-test, two tailed, **p = 0.0033, ***p < 0.0001). **Fig. S10.** No functional loss of RGCs in 5 weeks Dex-injected mice. C57BL/6 J mice were periocularly injected with Veh or Dex for 5 weeks, and RGC functional loss was examined using PERG. A representative wave graphs is shown in Veh (**A**) and Dex (**B**) injected mice demonstrated no significant differences in PERG amplitudes and latencies between Veh and Dex-treated mice. **Fig. S11.** Astrocytes activation and immune cell infiltration during the early stages of Dex induced neurodegeneration. C57BL/6 J mice were periocularly injected with Veh or Dex for 5 weeks, and reactive astrocytes and immune cell infiltration was analyzed by immunostaining. (**A&B**) Reactive astrocytes with increased expression of GFAP were observed in ONH cross sections in 5 weeks Dex-injected mice compared to Veh-injected mice. (**B&C**) Moderately increased number of F4/80^+^ macrophage-like cells in 5 weeks Dex-injected mice compared to Veh-injected mice. Data are shown as mean ± SD (n = 3 in each group, unpaired t-test, two tailed, *p = 0.04, ns = not significant *p* = 0.2). **Fig. S12.** Presence of axonal degeneration in entire length of optic nerve in Dex-treated mice. C57BL/6 J mice were periocularly injected with Veh or Dex for 10 weeks and axonal degeneration along the entire optic nerve including proximal, center and distal regions was examined using PPD staining. The representative images shown in Veh and Dex-injected mice demonstrated significant differences in axonal degeneration along the entire optic nerve between Veh and Dex- injected mice.

## Data Availability

All the data generated in the current studies will be made readily available from the corresponding authors upon reasonable request.

## Abbreviations

POAG: Primary open angle glaucoma
IOP: Intraocular pressure
TM: Trabecular meshwork
RGCs: Retinal ganglion cells
ON: Optic nerve
ONH: Optic nerve head
PPD: Paraphenylenediamide
Dex: Dexamethasone-21-acetate
Veh: Vehicle
ECM: Extracellular matrix
CTB: Cholera toxin B
CNS: Central nervous system
GFAP: Glial fibrillary acidic protein
OHT: Ocular hypertension
SC: Superior Colliculus
PERG: Pattern electroretinography
AH: Aqueous humor
H&E: Hematoxylin and Eosin
GC: Glucocorticoid
TEM: Transmission electron microscopy
TNF-α: Tumor Necrosis Factor

## References

[1] Kingman S (2004). Glaucoma is second leading cause of blindness globally. Bull World Health Organ.

[2] Tham YC, Li X, Wong TY, Quigley HA, Aung T, Cheng CY (2014). Global prevalence of glaucoma and projections of glaucoma burden through 2040: a systematic review and meta-analysis. Ophthalmology..

[3] Quigley HA, Broman AT (2006). The number of people with glaucoma worldwide in 2010 and 2020. Br J Ophthalmol.

[4] Foster A, Resnikoff S (2005). The impact of vision 2020 on global blindness. Eye (Lond).

[5] Kapetanakis VV, Chan MP, Foster PJ, Cook DG, Owen CG, Rudnicka AR (2016). Global variations and time trends in the prevalence of primary open angle glaucoma (POAG): a systematic review and meta-analysis. Br J Ophthalmol.

[6] Comparison of glaucomatous progression between untreated patients with normal-tension glaucoma and patients with therapeutically reduced intraocular pressures (1998). Collaborative Normal-tension Glaucoma study group. Am J Ophthalmol.

[7] The effectiveness of intraocular pressure reduction in the treatment of normal-tension glaucoma (1998). Collaborative Normal-Tension Glaucoma Study Group. Am J Ophthalmol.

[8] Garway-Heath DF, Crabb DP, Bunce C, Lascaratos G, Amalfitano F, Anand N (2015). Latanoprost for open-angle glaucoma (UKGTS): a randomised, multicentre, placebo-controlled trial. Lancet..

[9] Goel M, Picciani RG, Lee RK, Bhattacharya SK (2010). Aqueous humor dynamics: a review. Open Ophthalmol J.

[10] Roy Chowdhury U, Hann CR, Stamer WD, Fautsch MP (2015). Aqueous humor outflow: dynamics and disease. Invest Ophthalmol Vis Sci.

[11] Kwon YH, Fingert JH, Kuehn MH, Alward WL (2009). Primary open-angle glaucoma. N Engl J Med.

[12] Weinreb RN, Khaw PT (2004). Primary open-angle glaucoma. Lancet..

[13] Rohen JW, Lutjen-Drecoll E, Flugel C, Meyer M, Grierson I (1993). Ultrastructure of the trabecular meshwork in untreated cases of primary open-angle glaucoma (POAG). Exp Eye Res.

[14] Acott TS, Kelley MJ (2008). Extracellular matrix in the trabecular meshwork. Exp Eye Res.

[15] Araujo SV, Spaeth GL, Roth SM, Starita RJ (1995). A ten-year follow-up on a prospective, randomized trial of postoperative corticosteroids after trabeculectomy. Ophthalmology..

[16] Nouri-Mahdavi K, Brigatti L, Weitzman M, Caprioli J (1995). Outcomes of trabeculectomy for primary open-angle glaucoma. Ophthalmology..

[17] Gupta N, Yucel YH (2007). Glaucoma as a neurodegenerative disease. Curr Opin Ophthalmol.

[18] Overby DR, Clark AF (2015). Animal models of glucocorticoid-induced glaucoma. Exp Eye Res.

[19] Aihara M, Lindsey JD, Weinreb RN (2003). Experimental mouse ocular hypertension: establishment of the model. Invest Ophthalmol Vis Sci.

[20] Mabuchi F, Aihara M, Mackey MR, Lindsey JD, Weinreb RN (2003). Optic nerve damage in experimental mouse ocular hypertension. Invest Ophthalmol Vis Sci.

[21] Sappington RM, Carlson BJ, Crish SD, Calkins DJ (2010). The microbead occlusion model: a paradigm for induced ocular hypertension in rats and mice. Invest Ophthalmol Vis Sci.

[22] Zhang J, Li L, Huang H, Fang F, Webber HC, Zhuang P, et al. Silicone oil-induced ocular hypertension and glaucomatous neurodegeneration in mouse. Elife. 2019;8. 10.7554/eLife.45881 PMC6533060 31090540

[23] John SW, Smith RS, Savinova OV, Hawes NL, Chang B, Turnbull D (1998). Essential iris atrophy, pigment dispersion, and glaucoma in DBA/2J mice. Invest Ophthalmol Vis Sci.

[24] Anderson MG, Smith RS, Hawes NL, Zabaleta A, Chang B, Wiggs JL (2002). Mutations in genes encoding melanosomal proteins cause pigmentary glaucoma in DBA/2J mice. Nat Genet.

[25] Becker B, Mills DW (1963). Corticosteroids and intraocular pressure. Arch Ophthalmol.

[26] Armaly MF (1963). Effect of corticosteroids on intraocular pressure and fluid dynamics. Ii. The effect of dexamethasone in the glaucomatous eye. Arch Ophthalmol.

[27] Jones R, Rhee DJ (2006). Corticosteroid-induced ocular hypertension and glaucoma: a brief review and update of the literature. Curr Opin Ophthalmol.

[28] Armaly MF (1963). Effect of corticosteroids on intraocular pressure and fluid dynamics. I. the effect of dexamethasone in the Normal eye. Arch Ophthalmol.

[29] Becker B (1965). Intraocular Pressure Response to Topical Corticosteroids. Investig Ophthalmol.

[30] Clark AF, Wordinger RJ (2009). The role of steroids in outflow resistance. Exp Eye Res.

[31] Wordinger RJ, Clark AF (1999). Effects of glucocorticoids on the trabecular meshwork: towards a better understanding of glaucoma. Prog Retin Eye Res.

[32] Kasetti RB, Maddineni P, Millar JC, Clark AF, Zode GS (2017). Increased synthesis and deposition of extracellular matrix proteins leads to endoplasmic reticulum stress in the trabecular meshwork. Sci Rep.

[33] Kasetti RB, Maddineni P, Patel PD, Searby C, Sheffield VC, Zode GS (2018). Transforming growth factor beta2 (TGFbeta2) signaling plays a key role in glucocorticoid-induced ocular hypertension. J Biol Chem.

[34] Clark AF, Wilson K, de Kater AW, Allingham RR, McCartney MD (1995). Dexamethasone-induced ocular hypertension in perfusion-cultured human eyes. Invest Ophthalmol Vis Sci.

[35] Clark AF, Wilson K, McCartney MD, Miggans ST, Kunkle M, Howe W (1994). Glucocorticoid-induced formation of cross-linked actin networks in cultured human trabecular meshwork cells. Invest Ophthalmol Vis Sci.

[36] Zhang X, Ognibene CM, Clark AF, Yorio T (2007). Dexamethasone inhibition of trabecular meshwork cell phagocytosis and its modulation by glucocorticoid receptor beta. Exp Eye Res.

[37] Whitlock NA, McKnight B, Corcoran KN, Rodriguez LA, Rice DS (2010). Increased intraocular pressure in mice treated with dexamethasone. Invest Ophthalmol Vis Sci.

[38] Zode GS, Sharma AB, Lin X, Searby CC, Bugge K, Kim GH (2014). Ocular-specific ER stress reduction rescues glaucoma in murine glucocorticoid-induced glaucoma. J Clin Invest.

[39] Li G, Lee C, Agrahari V, Wang K, Navarro I, Sherwood JM (2019). In vivo measurement of trabecular meshwork stiffness in a corticosteroid-induced ocular hypertensive mouse model. Proc Natl Acad Sci U S A.

[40] Patel GC, Phan TN, Maddineni P, Kasetti RB, Millar JC, Clark AF (2017). Dexamethasone-induced ocular hypertension in mice: effects of Myocilin and route of administration. Am J Pathol.

[41] Zode GS, Kuehn MH, Nishimura DY, Searby CC, Mohan K, Grozdanic SD (2011). Reduction of ER stress via a chemical chaperone prevents disease phenotypes in a mouse model of primary open angle glaucoma. J Clin Invest.

[42] Millar JC, Clark AF, Pang IH (2011). Assessment of aqueous humor dynamics in the mouse by a novel method of constant-flow infusion. Invest Ophthalmol Vis Sci.

[43] Maddineni P, Kasetti RB, Zode GS (2018). Methods for analyzing endoplasmic reticulum stress in the trabecular meshwork of Glaucoma models. Methods Mol Biol.

[44] Rueden CT, Schindelin J, Hiner MC, DeZonia BE, Walter AE, Arena ET (2017). ImageJ2: ImageJ for the next generation of scientific image data. BMC Bioinformatics.

[45] Chou TH, Bohorquez J, Toft-Nielsen J, Ozdamar O, Porciatti V (2014). Robust mouse pattern electroretinograms derived simultaneously from each eye using a common snout electrode. Invest Ophthalmol Vis Sci.

[46] Morrison JC, Cepurna Ying Guo WO, Johnson EC (2011). Pathophysiology of human glaucomatous optic nerve damage: insights from rodent models of glaucoma. Exp Eye Res.

[47] Quigley HA, Addicks EM (1981). Regional differences in the structure of the lamina cribrosa and their relation to glaucomatous optic nerve damage. Arch Ophthalmol.

[48] Weinreb RN, Leung CK, Crowston JG, Medeiros FA, Friedman DS, Wiggs JL (2016). Primary open-angle glaucoma. Nat Rev Dis Primers.

[49] Quigley HA, Addicks EM (1980). Chronic experimental glaucoma in primates. II. Effect of extended intraocular pressure elevation on optic nerve head and axonal transport. Invest Ophthalmol Vis Sci.

[50] Hernandez MR (2000). The optic nerve head in glaucoma: role of astrocytes in tissue remodeling. Prog Retin Eye Res.

[51] Fukuchi T, Sawaguchi S, Hara H, Shirakashi M, Iwata K (1992). Extracellular matrix changes of the optic nerve lamina cribrosa in monkey eyes with experimentally chronic glaucoma. Graefes Arch Clin Exp Ophthalmol.

[52] Neufeld AH, Liu B (2003). Glaucomatous optic neuropathy: when glia misbehave. Neuroscientist..

[53] Tamm ER, Ethier CR, Lasker IIA (2017). Glaucomatous Neurodegeneration P. biological aspects of axonal damage in glaucoma: a brief review. Exp Eye Res.

[54] Quigley HA, Anderson DR (1977). Distribution of axonal transport blockade by acute intraocular pressure elevation in the primate optic nerve head. Invest Ophthalmol Vis Sci.

[55] Howell GR, Libby RT, Jakobs TC, Smith RS, Phalan FC, Barter JW (2007). Axons of retinal ganglion cells are insulted in the optic nerve early in DBA/2J glaucoma. J Cell Biol.

[56] Crish SD, Sappington RM, Inman DM, Horner PJ, Calkins DJ (2010). Distal axonopathy with structural persistence in glaucomatous neurodegeneration. Proc Natl Acad Sci U S A.

[57] Sihota R, Konkal VL, Dada T, Agarwal HC, Singh R (2008). Prospective, long-term evaluation of steroid-induced glaucoma. Eye (Lond)..

[58] Bollaerts I, Van Houcke J, Beckers A, Lemmens K, Vanhunsel S, De Groef L (2019). Prior exposure to Immunosuppressors sensitizes retinal microglia and accelerates optic nerve regeneration in Zebrafish. Mediat Inflamm.

[59] Johnson EC, Jia L, Cepurna WO, Doser TA, Morrison JC (2007). Global changes in optic nerve head gene expression after exposure to elevated intraocular pressure in a rat glaucoma model. Invest Ophthalmol Vis Sci.

[60] Hernandez MR, Miao H, Lukas T (2008). Astrocytes in glaucomatous optic neuropathy. Prog Brain Res.

[61] Wang R, Seifert P, Jakobs TC (2017). Astrocytes in the optic nerve head of glaucomatous mice display a characteristic reactive phenotype. Invest Ophthalmol Vis Sci.

[62] Harder JM, Braine CE, Williams PA, Zhu X, MacNicoll KH, Sousa GL (2017). Early immune responses are independent of RGC dysfunction in glaucoma with complement component C3 being protective. Proc Natl Acad Sci U S A.

[63] Williams PA, Braine CE, Kizhatil K, Foxworth NE, Tolman NG, Harder JM (2019). Inhibition of monocyte-like cell extravasation protects from neurodegeneration in DBA/2J glaucoma. Mol Neurodegener.

[64] Buckingham BP, Inman DM, Lambert W, Oglesby E, Calkins DJ, Steele MR (2008). Progressive ganglion cell degeneration precedes neuronal loss in a mouse model of glaucoma. J Neurosci.

[65] Dengler-Crish CM, Smith MA, Inman DM, Wilson GN, Young JW, Crish SD (2014). Anterograde transport blockade precedes deficits in retrograde transport in the visual projection of the DBA/2J mouse model of glaucoma. Front Neurosci.

[66] Howell GR, Soto I, Zhu X, Ryan M, Macalinao DG, Sousa GL (2012). Radiation treatment inhibits monocyte entry into the optic nerve head and prevents neuronal damage in a mouse model of glaucoma. J Clin Invest.

[67] El-Danaf RN, Huberman AD (2015). Characteristic patterns of dendritic remodeling in early-stage glaucoma: evidence from genetically identified retinal ganglion cell types. J Neurosci.

[68] Risner ML, Pasini S, Cooper ML, Lambert WS, Calkins DJ (2018). Axogenic mechanism enhances retinal ganglion cell excitability during early progression in glaucoma. Proc Natl Acad Sci U S A.

[69] Jakobs TC, Libby RT, Ben Y, John SW, Masland RH (2005). Retinal ganglion cell degeneration is topological but not cell type specific in DBA/2J mice. J Cell Biol.

