# Supplementary material for: CNS axonal degeneration and transport deficits at the optic nerve head precede structural and functional loss of retinal ganglion cells in a mouse model of glaucoma
Source: Mol Neurodegener. 2020 Aug 27;15:48. doi: 10.1186/s13024-020-00400-9 (PMC7457267; doi:10.1186/s13024-020-00400-9)
Supplement: Supplementary file 1 — Additional file 1: Fig. S1. Dex-induced IOP elevation is higher during the night-time. C57BL/6 J mice were periocularly injected with Veh or Dex in both eyes, and IOPs were monitored weekly in the dark during the night time under isoflurane anesthetic conditions. Dex injections lead to sustained and significant IOP elevation and the observed mean difference of Dex-induced IOP is more pronounced at night compared to daytime IOP. Data are shown as mean ± SD (n = 8 in each group, 2-WAY ANOVA with multiple comparison, ***p = 0.0002, #p < 0.0001). Fig. S2. Effect of periocular administration of Dex on body weight in mice. 3-months old C57BL/6 J mice were periocularly injected with Veh or Dex weekly for 10 weeks, and body weight was monitored before and every week after injections. No significant difference in body weight between Veh and Dex-injected mice was observed. Both Veh and Dex-treated mice continued to gain weight as expected. Data are shown as mean ± SD (n = 5 in each group, 2-WAY ANOVA with multiple comparison). Fig. S3. Dex induced ECM proteins deposition and cytoskeleton changes in mouse TM. Densitometry analysis confirmed significantly increased deposition of major ECM proteins FN, ColI and laminin and also increased cytoskeleton markers including alpha smooth muscle (SMA) and phalloidin in the TM tissues of 10 weeks Dex injected mice compared to Veh injected mice. Data are shown as mean ± SD (n = 4 or 6 in each group, unpaired t-test, two tailed, **p = 0.002, #p < 0.0001). Fig. S4 Effect of periocular Dex administration on ocular tissues. Slit lamp imaging and H&E staining was performed in 10 weeks Veh or Dex-injected mice. Slit lamp images (left panel) demonstrate no ocular abnormalities. H & E staining reveals no changes in gross morphology of iridocorneal angles as well as no other ocular structural abnormalities in Veh or Dex-injected eyes (n = 6). Fig. S5. Effect of Dex-induced OHT on retinal layers. A) Representative images of H&E staining on retinas [file 13024_2020_400_MOESM1_ESM.docx]

**Supplementary Figures**

**Maddineni et al**

**
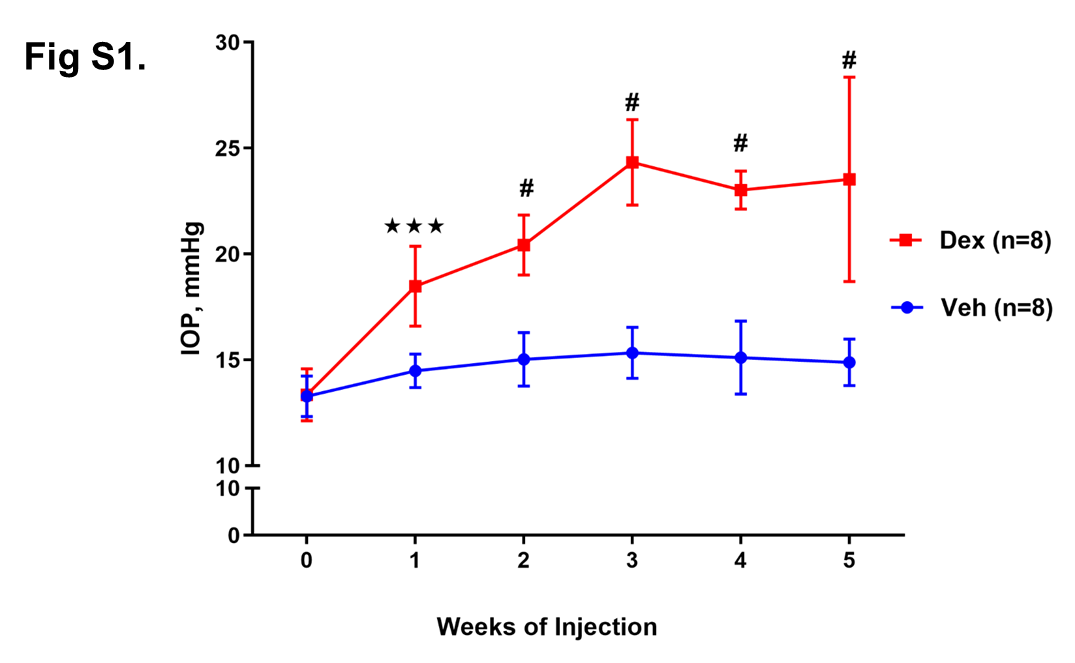
**

**S1.** Dex-induced IOP elevation is higher during the night-time. C57BL/6J mice were periocularly injected with Veh or Dex in both eyes, and IOPs were monitored weekly in the dark during the night time under isoflurane anesthetic conditions. Dex injections lead to sustained and significant IOP elevation and the observed mean difference of Dex-induced IOP is more pronounced at night compared to daytime IOP. Data are shown as mean±SD (n=8 in each group, 2-WAY ANOVA with multiple comparison, ***p=0.0002, #p<0.0001).

**
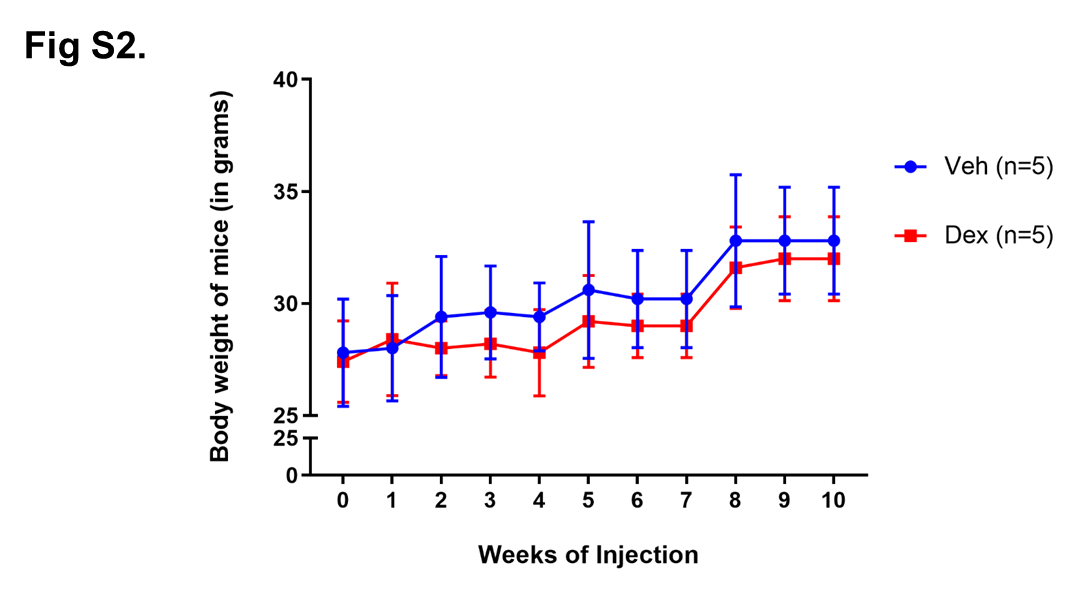
**

**S2.** Effect of periocular administration of Dex on body weight in mice. 3-months old C57BL/6J mice were periocularly injected with Veh or Dex weekly for 10 weeks, and body weight was monitored before and every week after injections. No significant difference in body weight between Veh and Dex-injected mice was observed. Both Veh and Dex-treated mice continued to gain weight as expected. Data are shown as mean±SD (n=5 in each group, 2-WAY ANOVA with multiple comparison).


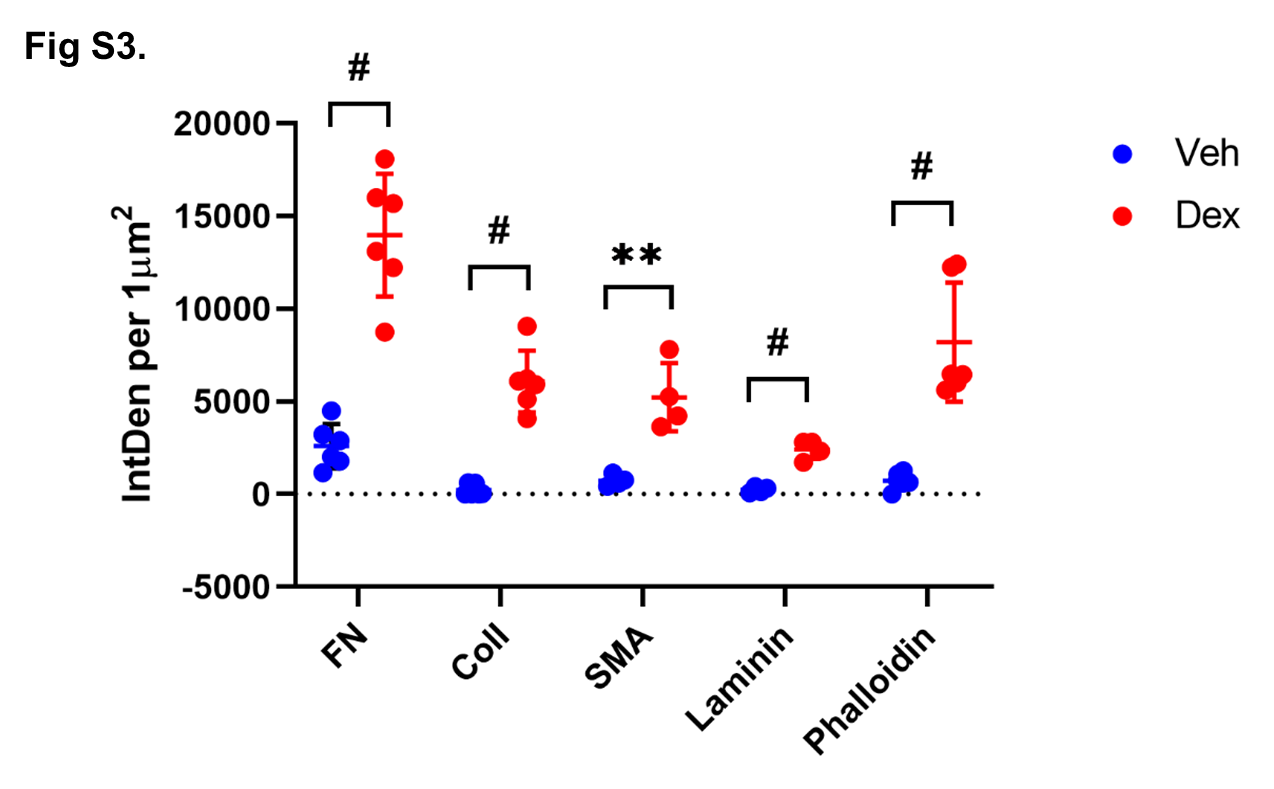


**S3.** Dex induced ECM proteins deposition and cytoskeleton changes in mouse TM. Densitometry analysis confirmed significantly increased deposition of major ECM proteins FN, ColI and laminin and also increased cytoskeleton markers including alpha smooth muscle (SMA) and phalloidin in the TM tissues of 10 weeks Dex injected mice compared to Veh injected mice. Data are shown as mean±SD (n=4 or 6 in each group, unpaired t-test, two tailed, **p=0.002, #p<0.0001).


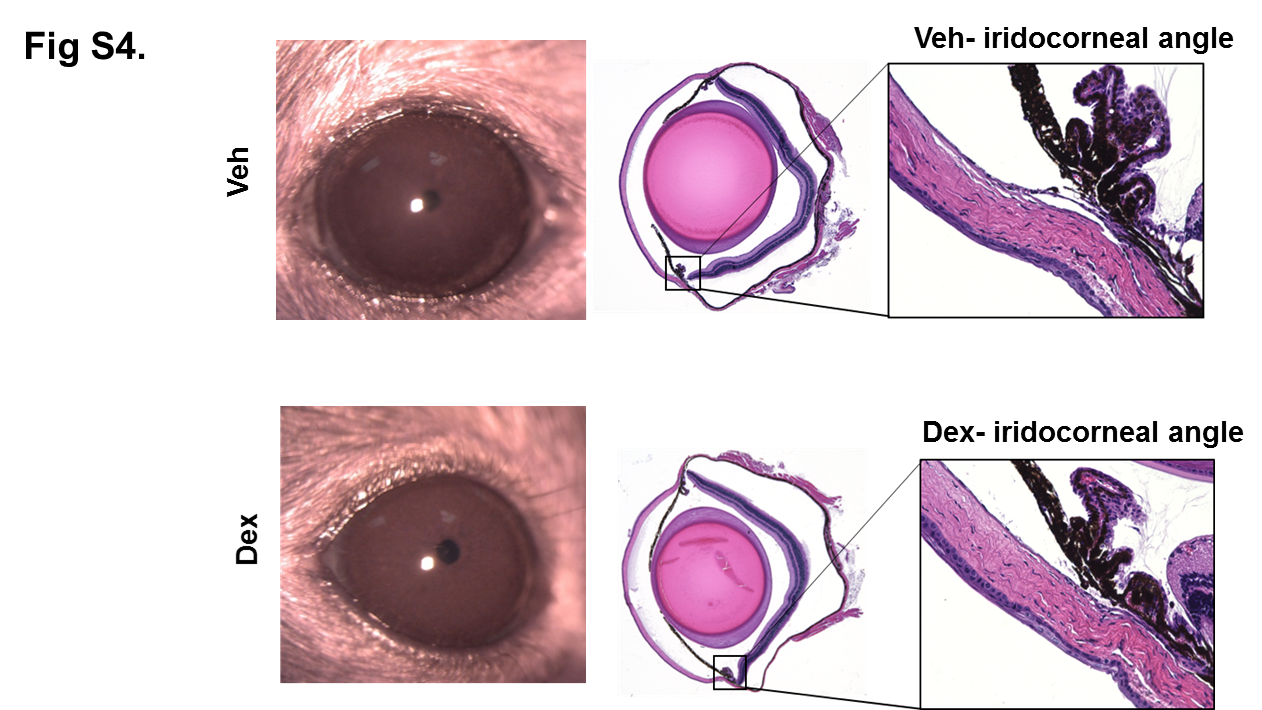


**S4.** Effect of periocular Dex administration on ocular tissues. Slit lamp imaging and H&E staining was performed in 10 weeks Veh or Dex-injected mice. Slit lamp images (left panel) demonstrate no ocular abnormalities. H & E staining reveals no changes in gross morphology of iridocorneal angles as well as no other ocular structural abnormalities in Veh or Dex-injected eyes (n=6).

**S5.** Effect of Dex-induced OHT on retinal layers. **A**) Representative images of H&E staining on retinas of 10 weeks Veh and Dex-injected mice. **B**) IPL layer thickness was measured and represented graphically. Except for the cell loss in the RGC layer, there were no gross morphological differences in other retinal layers including IPL between Veh and Dex-injected eyes (n=3 in each group, ns=not significant, p=0.5).

**
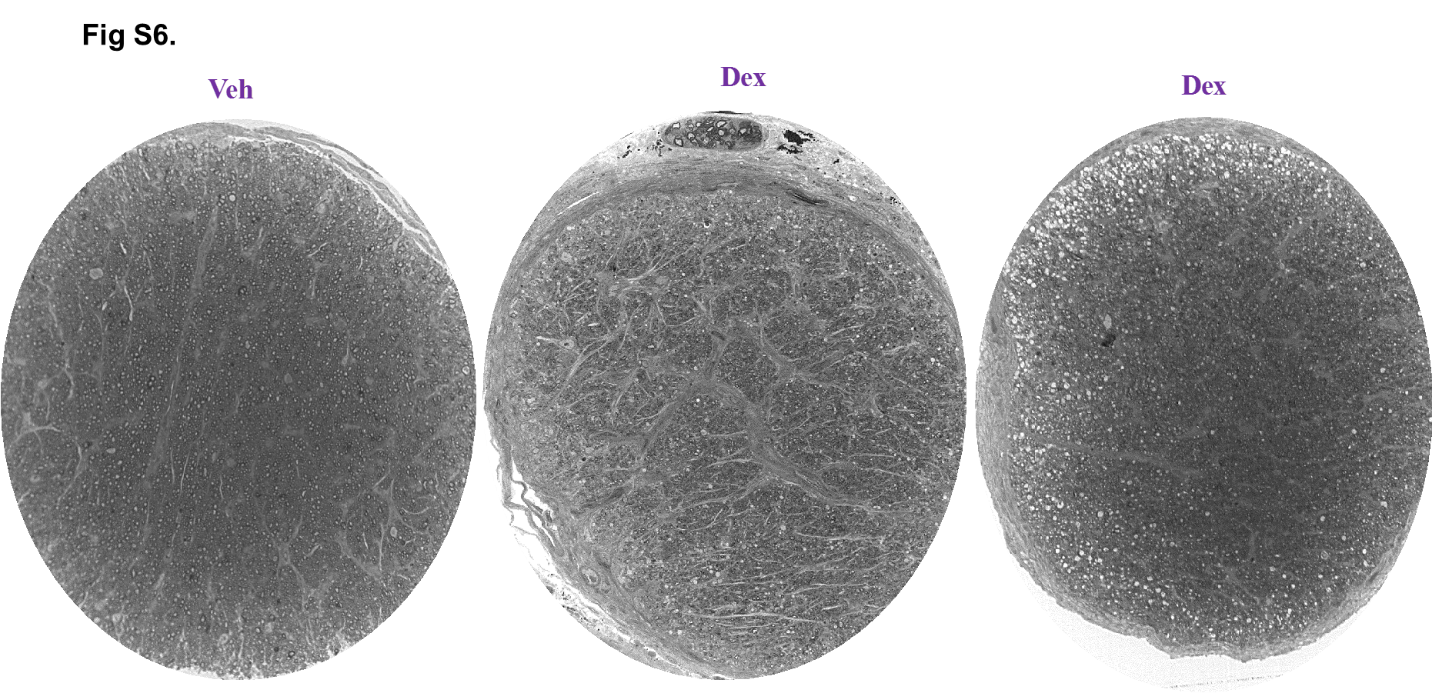
**

**Fig S6.** Optic nerve axonal degeneration in Veh and Dex-injected mice. PPD staining was performed on cross sections of optic nerves collected from 10 weeks Veh or Dex-injected mice. Optic nerves from Dex-injected mice show severe axonal degeneration with extensive glial scar formation and reduced number of healthy axons with severe vacuolization patterns compared to optic nerves from Veh injected mice (n=6 in each group).


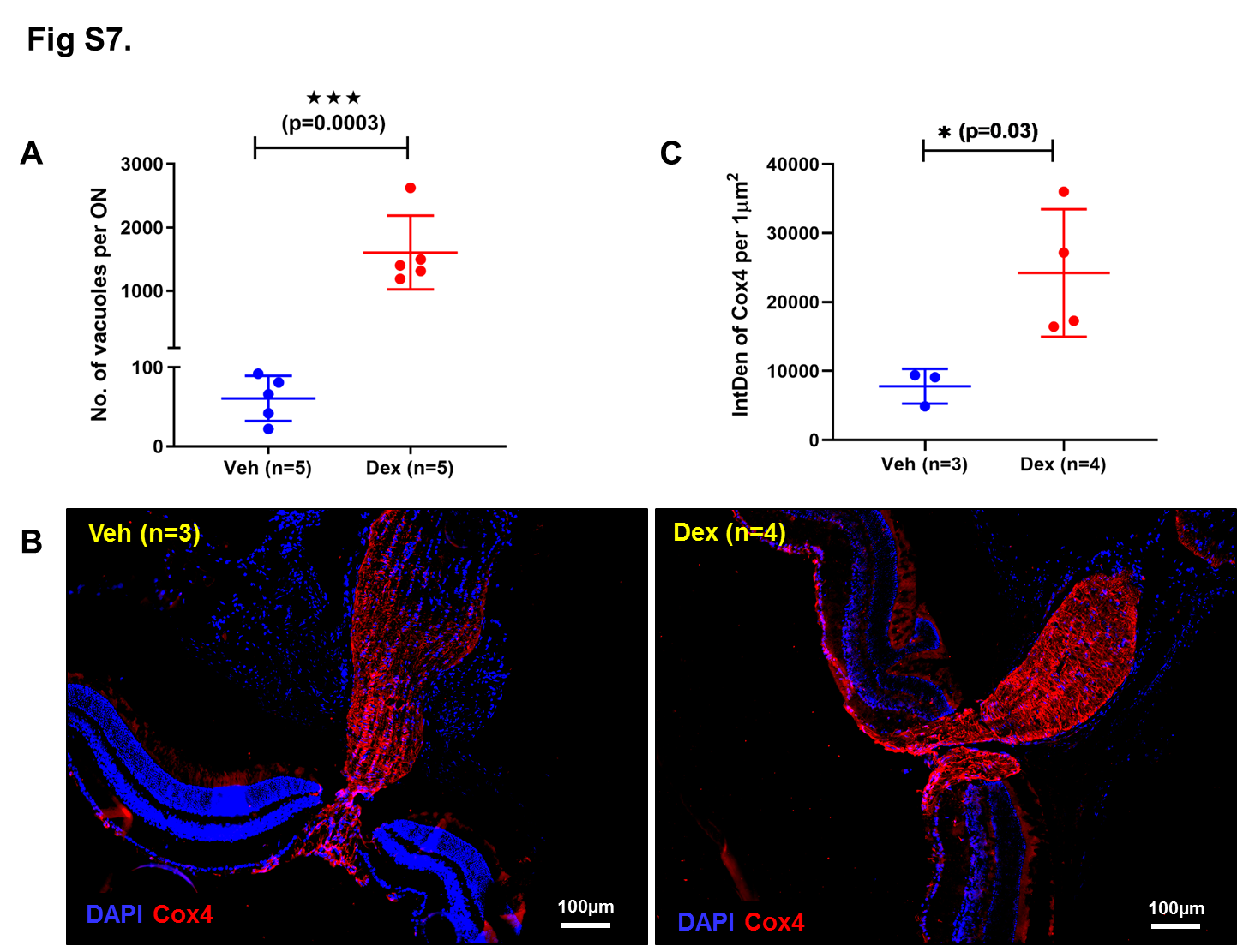


**Fig S7**. Presence of vacuoles and accumulation of mitochondria in association with Dex induced axonal degeneration. C57BL/6J mice were periocularly injected with Veh or Dex for 10 weeks, and (**A**) vacuoles were counted from PPD stained cross sections of ON. (**B&C**) Mitochondrial accumulation was evaluated by immunostaining with Cox4 and Cox4 florescent intensity was measured and shown graphically. The representative dot plots demonstrated significantly increased number of vacuoles as well as mitochondrial accumulation in Dex-treated mice compared to Veh-treated mice (**A&C**). Data are shown as mean±SD (n=3-5 in each group, unpaired t-test, two tailed, *p=0.03, ***p=0.0003).


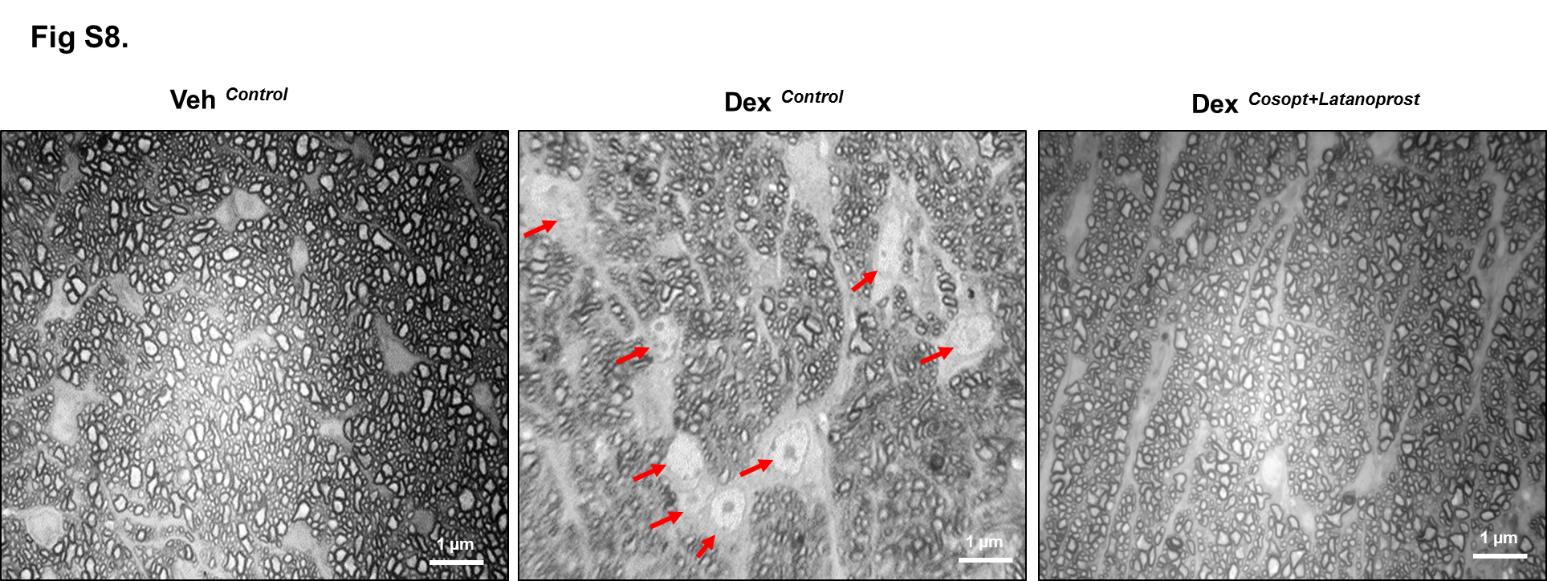


**Fig S8.** Reduction of elevated IOP prevents immune cells infiltration in mouse optic nerve. Enlarged images of PPD stained cross sections of optic nerve from Dex*^Control^* mice showed presence of infiltrated immune cells in the degenerated optic nerves. Reduction of IOP prevents immune cells infiltration in optic nerve of Dex*^Cosopt+Latanoprost^* mice. (n=3, the arrow heads pointed toward infiltrated immune cells)


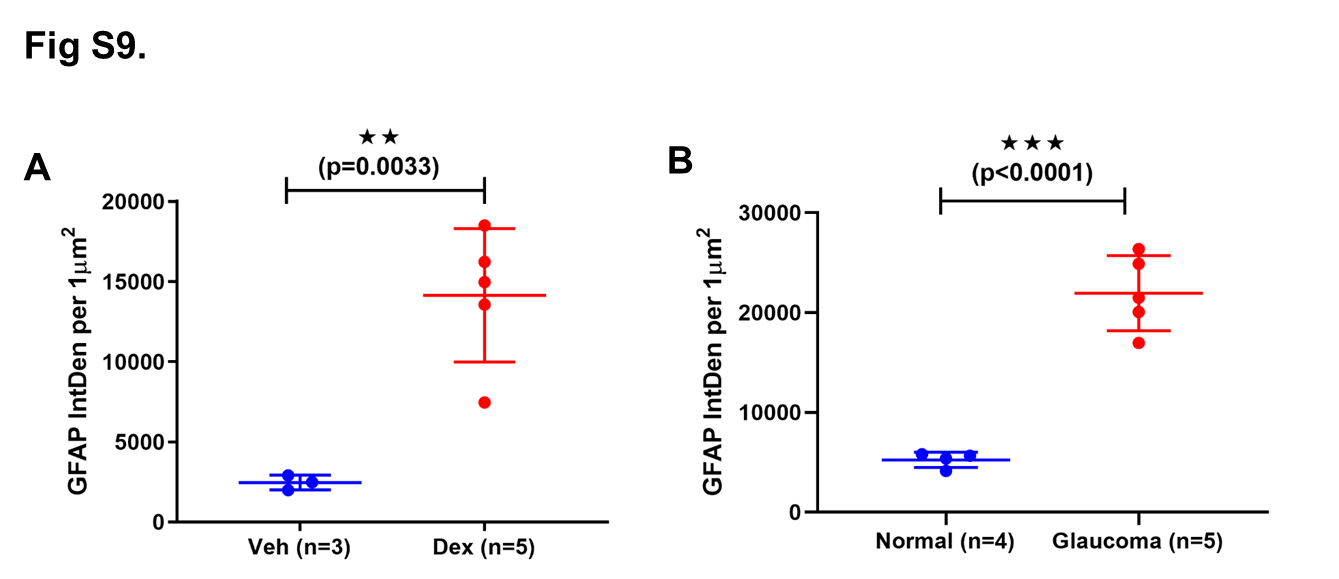


**Fig S9.** Presence of reactive astrocytes in the glaucomatous ONH. A representative dot plots showed significant increase in activation of astrocytes at ONH region of 10 weeks Dex injected mice (**A**) and in human glaucomatous ONH region (**B**). Data are shown as mean±SD (unpaired t-test, two tailed, **p=0.0033, ***p<0.0001).

**
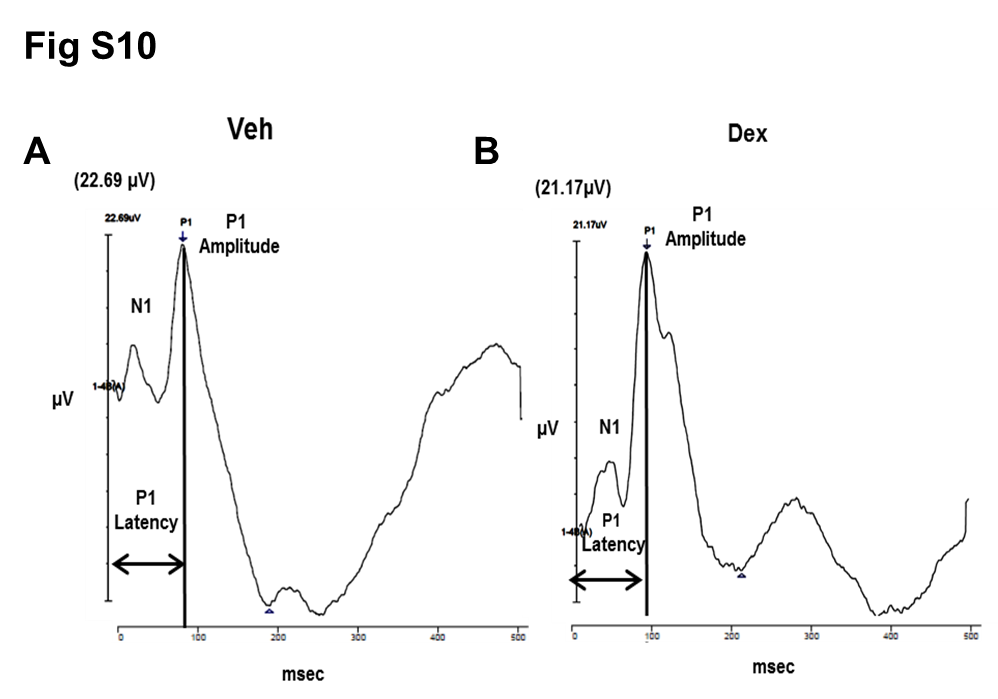
**

**Fig S10.** No functional loss of RGCs in 5 weeks Dex-injected mice. C57BL/6J mice were periocularly injected with Veh or Dex for 5 weeks, and RGC functional loss was examined using PERG. A representative wave graphs is shown in Veh (**A**) and Dex (**B**) injected mice demonstrated no significant differences in PERG amplitudes and latencies between Veh and Dex-treated mice.

**Fig S11.**  Astrocytes activation and immune cell infiltration during the early stages of Dex induced neurodegeneration. C57BL/6J mice were periocularly injected with Veh or Dex for 5 weeks, and reactive astrocytes and immune cell infiltration was analyzed by immunostaining. (**A&B**) Reactive astrocytes with increased expression of GFAP were observed in ONH cross sections in 5 weeks Dex-injected mice compared to Veh-injected mice. (**B&C**) Moderately increased number of F4/80^+^ macrophage-like cells in 5 weeks Dex-injected mice compared to Veh-injected mice. Data are shown as mean±SD (n=3 in each group, unpaired t-test, two tailed, *p=0.04, ns=not significant p=0.2).

**
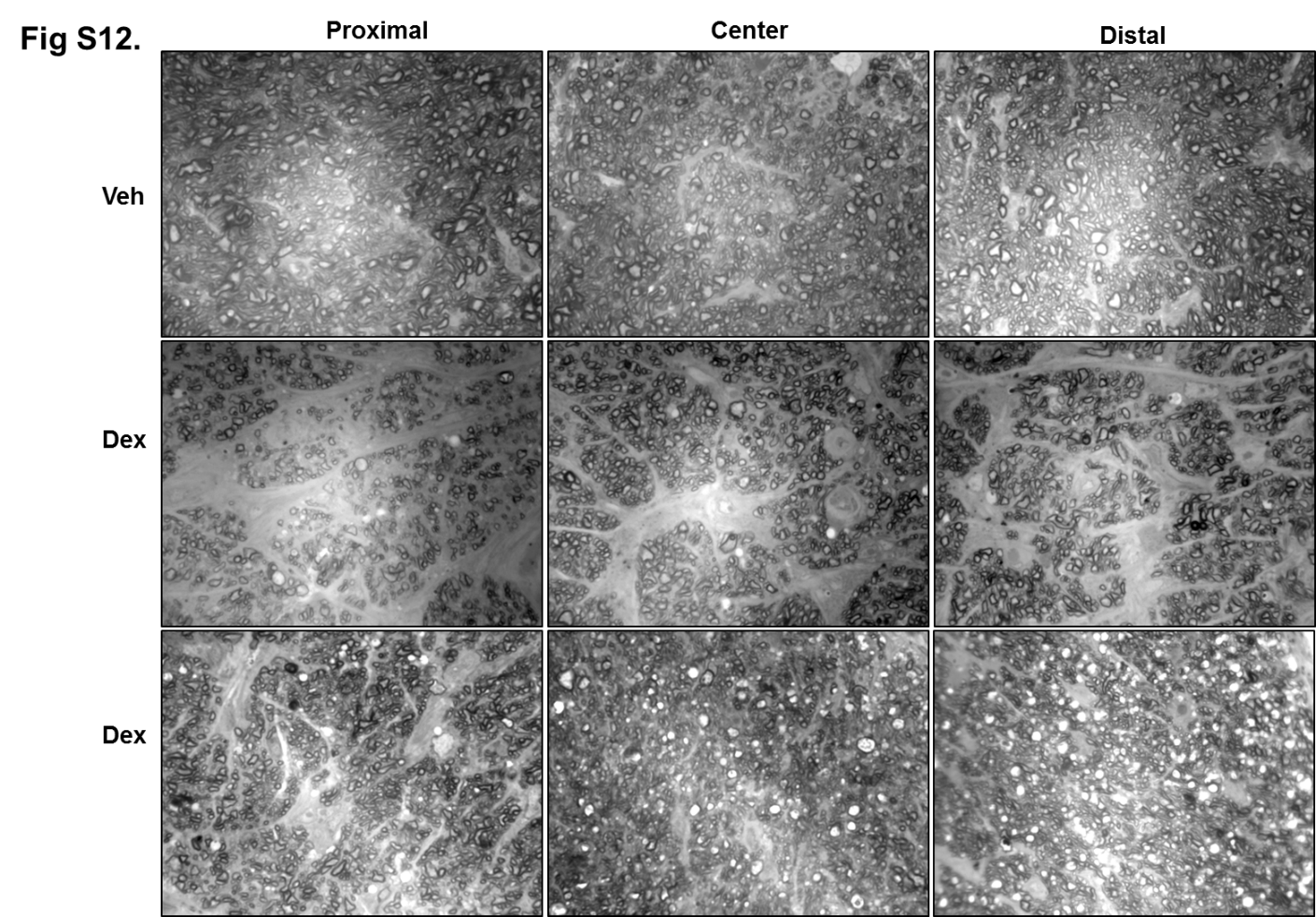
**

**Fig S12.** Presence of axonal degeneration in entire length of optic nerve in Dex-treated mice. C57BL/6J mice were periocularly injected with Veh or Dex for 10 weeks and axonal degeneration along the entire optic nerve including proximal, center and distal regions was examined using PPD staining. The representative images shown in Veh and Dex-injected mice demonstrated significant differences in axonal degeneration along the entire optic nerve between Veh and Dex- injected mice.
